# Mammalian non-CG methylations are conserved and cell-type specific and may have been involved in the evolution of transposon elements

**DOI:** 10.1038/srep32207

**Published:** 2016-08-30

**Authors:** Weilong Guo, Michael Q. Zhang, Hong Wu

**Affiliations:** 1 The MOE Key Laboratory of Cell Proliferation and Differentiation, School of Life Sciences, Peking University, Beijing, People’s Republic of China; 2 Department of Biological Sciences, Center for Systems Biology, The University of Texas at Dallas, Richardson, United States of America; 3 Bioinformatics Division, TNLIST and Center for Synthetic & Systems Biology, Tsinghua University, Beijing, People’s Republic of China

## Abstract

Although non-CG methylations are abundant in several mammalian cell types, their biological significance is sparsely characterized. We gathered 51 human and mouse DNA methylomes from brain neurons, embryonic stem cells and induced pluripotent stem cells, primordial germ cells and oocytes. We utilized an unbiased sub-motif prediction method and reported CW as the representative non-CG methylation context, which is distinct from CC methylation in terms of sequence context and genomic distribution. A two-dimensional comparison of non-CG methylations across cell types and species was performed. Unambiguous studies of sequence preferences and genomic region enrichment showed that CW methylation is cell-type specific and is also conserved between humans and mice. In brain neurons, it was found that active long interspersed nuclear element-1 (LINE-1) lacked CW methylations but not CG methylations. Coincidentally, both human Alu and mouse B1 elements preferred high CW methylations at specific loci during their respective evolutionary development. Last, the strand-specific distributions of CW methylations in introns and long interspersed nuclear elements are also cell-type specific and conserved. In summary, our results illustrate that CW methylations are highly conserved among species, are dynamically regulated in each cell type, and are potentially involved in the evolution of transposon elements.

In mammals, CG methylations have been extensively studied for decades and have been found to be conserved^1^,^2^ and dynamically regulated in development^3^. CG methylations play important roles in regulating gene transcription^4^ and silencing transposon element (TE) activities^5^. Mammalian non-CG methylations, also known as CH (H can be A, C, T) methylations, were reported to be abundant only in specific cell types and low in most somatic cell types^6^,^7^,^8^. Although CH methylation (mCH) has been well studied in *Arabidopsis*^9^, it is still unclear whether mCH has a similar function in mammals^10^. Taking advantage of the accumulating DNA methylomes across multiple cell types and species, we aimed to shed light on the potential functions of mammalian mCH.

Based on current knowledge, mammalian mCH-enriched cell types can be categorized into two main categories: brain neurons^11^,^12^ and germline cells. The mCH-enriched germline cells consist of embryonic stem cells (ESCs)^13^, induced pluripotent stem cells (iPSCs)^14^, oocytes^15^,^16^,^17^, and male and female primordial germ cells (PGCs)^18^,^19^,^20^,^21^,^22^. Unlike in oocytes^15^, mCH rarely occurs in sperm cells^23^,^24^. Most differentiated cells, such as fibroblast cells^13^, blood cells^7^, are low in mCH. A recent study reported that mCH was abundant in myocytes^25^, extending our understanding of somatic mCH. Although several reports have described potential roles for mCH, the explicit biological functions of mCH remain a mystery^6^.

Comparing mCH across different cell types or species is an efficient way to characterize such modifications. Chen and colleagues performed inter-sample comparisons of mCH in human ESCs and demonstrated that mCH is conserved in TACAG contexts^26^. *Lister et al*. compared human ESCs and iPSCs, showing that mCH is increased during reprogramming but in an incomplete manner^14^. Subsequently, *Ziller et al*. performed a comparison of a panel of human DNA methylomes^7^ and confirmed the abundance of mCH in pluripotent cell types and found that mCH was significantly dependent on DNMT3 expression. *Varley et al*. generated 82 human methylomes and found that brain mCH is similar among individuals but has a different motif as in ES^27^. By comparing brain methylomes from humans and mice, *Lister* and colleagues illustrated that mCH is enriched in neurons and glias and the neuronal mCH is also highly conserved between the species^12^. Until now, there have been no studies investigating mCH across both cell types and species at the same time.

We investigated mCH by collecting 51 mCH-enriched DNA methylomes in both humans and mice using data previously published by multiple groups. In this cohort, the cell types included brain neurons, ESCs/iPSCs, oocytes, and male and female PGCs. Our previous study showed that the two contexts CHG and CHH are not necessary to be studied separately in human pluripotent cells^28^. We designed a computational method to predict the most significant bi-partition of the motif of highly methylated CH sites. Interestingly, almost all of the samples support CW and CC as the most independent sub-context. Context and spatial studies demonstrated that CW is the representative context for mCH. Our unsupervised clustering based on sequence preferences revealed that mCW is more closely related among cell types than among species. This result extended our understanding of mCW as a dynamically regulated DNA modification within different cell types, which is also highly conserved among species. Furthermore, we evaluated mCW enrichment in genes and in TEs, uncovering features of conservation and cell-type specificities.

Closer inspections of mCW distribution led us to several novel findings. In brain neurons, long interspersed nuclear element-1 (LINE-1) lacks mCW, especially young LINE-1. Simple repeats are enriched with mCW in all cell types but are particularly pronounced in PGCs. Coincidentally, both human Alu elements and murine B1 elements showed several loci preferred higher mCW during evolution, extending current knowledge beyond their CG methylation (mCG) patterns^29^,^30^. Additionally, we found a peak of mCG at the promoter of young LINE-1 elements in PGCs, but not in other cell types. Our previous finding that intronic mCH was strand-skewed in human ES cells^28^ was also found in mouse ES cells. Further results revealed strand-specific distributions of mCW in certain TEs that are shared by the two species. In general, our work has advanced the knowledge of mammalian non-CG methylations and provided interesting clues for future investigations.

## Results

### mCH abundance DNA methylomes across species and cell types

To study mammalian mCH, we collected 51 bisulfite-sequencing libraries for both humans and mice (Table 1; Supplementary Table S1) and regenerated most of the methylomes from raw sequencing data using BS-Seeker2^31^. Investigated cell types included brain neurons, ESCs, iPSCs, oocytes, and male and female primordial germ cell (MPGCs and FPGCs, respectively). For each cell type, multiple samples from different groups were collected to eliminate inter-sample differences. In this cohort, average methylation levels of CG and CH among samples are discordant (Supplementary Fig. S1a). Also, the contributions of mCH to overall DNA methylation in the selected samples are inevitable (Supplementary Fig. S2).

### CW and CC are two different mCH contexts in mammals

**Figure 1:**
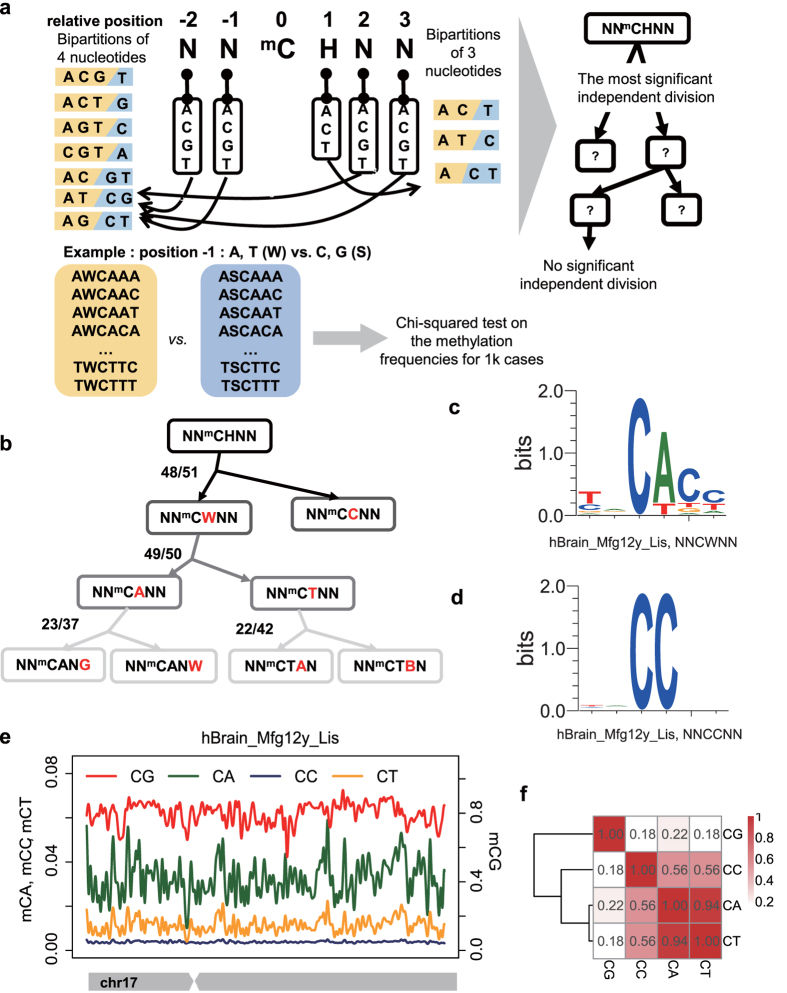
CW and CC are the major independent non-CG contexts. (**a**) Schematic of the MiDD (minimum dependence decomposition) method. Enumeration of all bipartitions at each base of the 6-mer (NN^m^CHNN) was conducted and the most significantly independent bipartition for at least half of the samples was adopted. The selection processes were performed for each sub-context recurrently until no significant bipartition was found. (**b**) The hierarchical motif tree of mCH built by MiDD. The numbers “n/m” beside the branches indicate that m samples report significant bipartition and that n samples report the represented division as the most significant bipartition. The red nucleotide indicates the position for bipartition. W = {A, T}. B = {C, G, T}. (**c,d**) The normalized logo plots for mCW (**c**) and mCC (**d**) in the sample *hBrain_Mfg12y_Lis*. (**e**) An example showing the methylation level profiles in four contexts (CA, CC, CG and CT) across chromosome 17. Lines are smoothed based on the average methylation levels in bins. Bin size, 20 k bp. (**f**) Heatmap showing the spatial correlation coefficients of the methylation levels among the four contexts (CA, CC, CG and CT) as in (**e**). Number in each cell, Pearson’s *r*. Distance is defined as (1−r^2^) for hierarchical clustering.

In plants, non-CG methylations have been studied separately in CHG and CHH contexts^1^. Our previous study showed that CHG and CHH methylations are highly correlated and not necessary to be separated in human pluripotent cells^28^. To unbiased identify any different sub-contexts of mCH in mammals, we developed a computational method named minimum dependence decomposition (MiDD) (see Methods). Considering the entropy at each position of previously reported mCH motifs^6^, we used the 6-mers (NN^m^CHNN, N = A, C, G, T) to characterize the sequence preference of mCH. Utilizing MiDD, the most significant bipartitions of the 6-mers were used for building a hierarchical motif tree for mammalian mCH (Fig. 1a). Interestingly, MiDD reported CW and CC as the most significantly independent bipartition of mammalian mCH (Fig. 1b). The average methylation levels of CW and CC are also discordant among all samples (Fig. S1b), and the conserved motifs of mCW and mCC were markedly different (Supplementary Fig. S3). For example, in the sample *hBrain_Mfg12y_Lis*, the mCW motif is TN^m^CACC (Fig. 1c), whereas the mCC motif is NN^m^CCNN (Fig. 1d).

To confirm our prediction from a spatial distribution perspective, we profiled the distributions of mCA, mCC, mCG and mCT levels across chromosomes. The distribution of mCC is apparently discordant with those of mCW and mCG (Fig. 1e–f). Although mCA and mCT were predicted to be the secondary significantly independent context decomposition of mCH (Fig. 1b), the chromosome-wide profiles of mCA and mCT were concordant (Fig. 1e, Supplementary Fig. S4) and highly correlated (Fig. 1f). In terms of bulk methylation levels, mCW was confirmed to be independent from mCG (Supplementary Fig. S5a), and mCHG and mCHH were generally concordant (Supplementary Figs S5e and S6), whereas mCC was weakly correlated with the other contexts (Supplementary Fig. S6). Considering all of these results, we decided to use the representative context CW in subsequent analysis.

### Context preferences of mCW are cell-type specific and conserved among species

**Figure 2:**
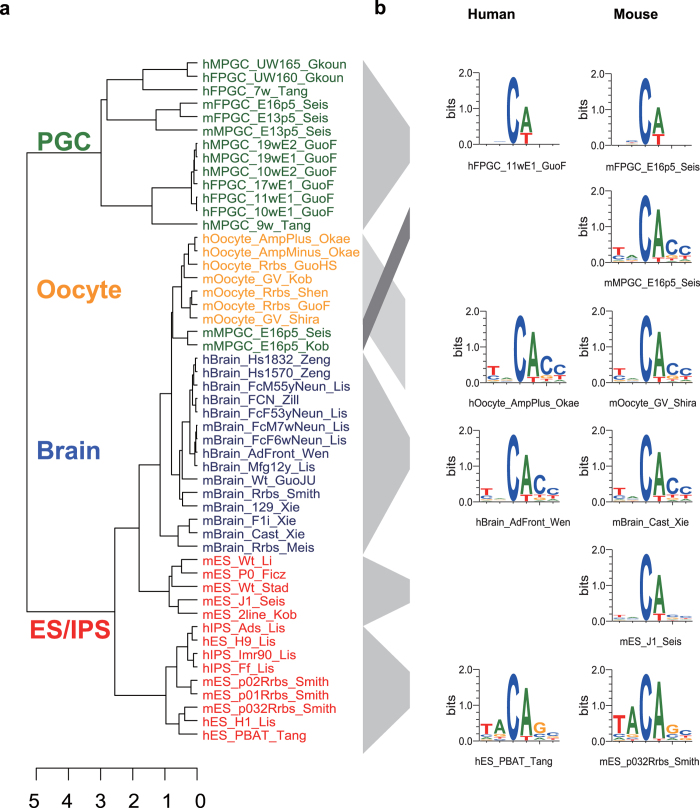
Comparison of the sequence preferences of mCW. (**a**) Unsupervised hierarchical clustering based on the ranking of the average methylation levels of 6-mers (NN^m^CWNN). Colours indicate cell types. Distance is defined as (1−ρ^2^), where ρ is Spearman’s correlation coefficient, measuring the similarity of the sequence preferences based on the bulk methylation levels of 6-mers for each sample pair. Clustering method, complete. (**b**) Logo plots of the normalized mCW motifs.

Currently, non-CG methylations are considered as context-dependent in mammals^26^,^28^. As mCW levels are different among samples, we used the ranks of the methylation levels of 6-mers (NN^m^CWNN) to represent the context preferences of mCW so that the samples are comparable. Thus to evaluate the motif similarities in mCW among samples, we performed an unsupervised clustering, where distance is defined according to Spearman’s rank correlation coefficients of the average methylation levels of the 6-mers (see Methods). To avoid the bias related to transcriptional activities or library types, we excluded annotated genetic regions, repeats and CpG islands when calculating average methylation levels. Interestingly, the results showed that the sequence preferences of mCW are more similar among cell types, rather than among species (Fig. 2a), indicating that the mCW motif is cell-type specific and is also conserved between humans and mice.

Based on the clustering results, the oocytes and brain neurons samples are grouped closely with similar motif, TN^m^CACC (Fig. 2b), which is in line with results from the previous studies^15^,^32^.

The ESC and iPSC samples are grouped into two sub-clusters (Fig. 2a) with different mCW motifs (Fig. 2b). Samples in one group are all from mice, harbouring the motif of NN^m^CAN. The other group has the motif TA^m^CAG, which includes all of the human ESCs/iPSCs and the mouse ESCs from *Smith et al*.^33^. Differences between human ESCs and mouse ESCs have been extensively discussed^34^. It has been proposed that mouse ESCs have the following two pluripotent states: a naïve ICM-like state and a primed pluripotent state. Human ESCs are in a primed pluripotent state^35^, and making it difficult to stably maintain a naïve human pluripotent stem cell line^34^. Our results indicate there may be distinct mCW signatures at different stages of pluripotency.

The majority of PGC samples fell into one group with a relatively weak motif of ^m^CA. The large distances between samples from different laboratories indicated a strong bench effect in gathering PGC samples or preparing libraries. Additionally, most of the MPGCs and all of the FPGCs are clustered together. Interestingly, two mouse MPGC samples, both of which are at E16.5, fell together within oocytes, with a motif of TN^m^CACC. Moreover, they were from different laboratories, ruling out the possibility of a bench effect. A previous study showed that MPGCs at E16.5 gained de novo CG methylation compared with earlier MPGC stages^21^. Our study provides additional evidence supporting the notion that the mouse MPGCs at E16.5 already gained epigenetic signatures comparable to mature oocytes.

Although DNMT3a, DNMT3b and DNMT3L have been reported to be responsible for non-CG methylations in mammals^7^,^15^, the three together are not necessary for a specific cell type^6^. As a reflection of biological orientation, mCW motifs are conserved between humans and mice, and are cell-type specific, indicating that mCW is elaborately maintained and regulated in different cell types.

### Enrichments of mCW are cell-type specific and conserved among species

**Figure 3:**
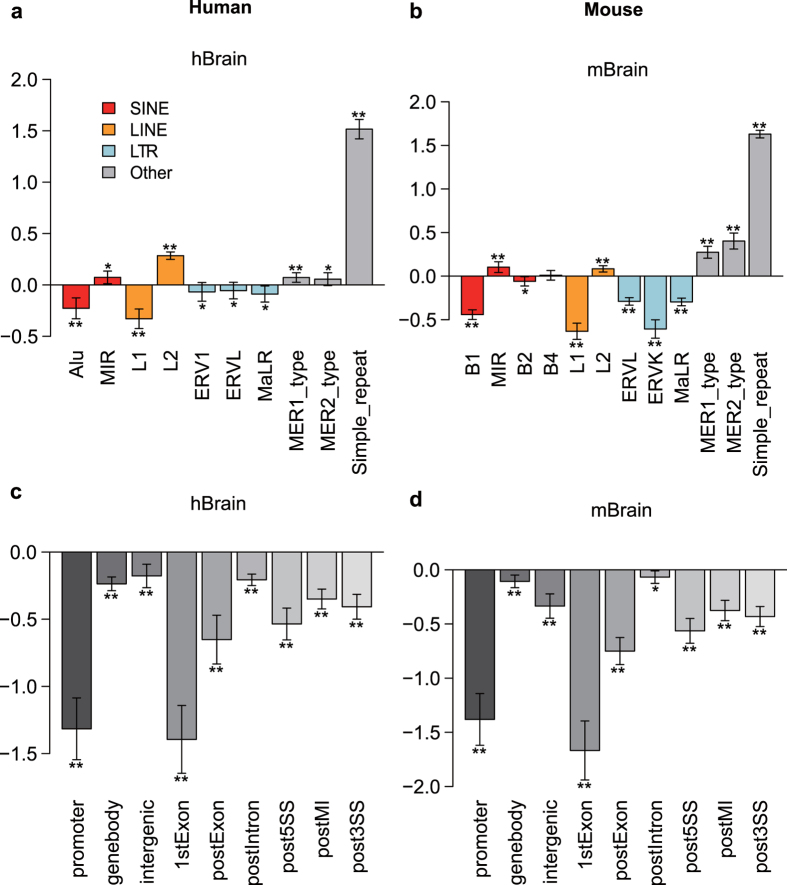
Enrichments of brain neuron mCW in genomic regions are consistent between humans and mice. Enrichment studies of the recurrent brain mCW sites in multiple samples in humans (**a,c**) and mice (**b,d**). The repeat elements investigated include SINEs, LINEs, LTRs and others (**a,b**). The gene related regions are separated as the promoter, gene body, intergenic regions, first exon (1stExon) and posterior exons (postExon), introns (postIntron), 5′ splicing site region (post5SS), middle intron (postMI) and 3′ splicing site region (post3SS). Y-axis, enrichment score, defined as the log_2_ fold changes between observed high mCW site count and expected high mCW site count. The mean (bar height), s.d. (error bar), and p-values (by two tailed *t* test; *p < 0.01; **p < 1e-6) are calculated based on the enrichment scores in all autosomes.

To investigate the possible functions of mCW, we quantified the enrichment of mCW in transposon elements, which make up approximately 45% of the human genome^36^. For each cell type, we used recurrent mCW sites among different samples versus randomly selected CW sites to calculate the fold enrichments of mCW in interrogated regions (see Methods). Interestingly, enrichments of brain neuronal mCW are consistent between humans (Fig. 3a) and mice (Fig. 3b). Brain LINE-1 elements and long terminal repeats (LTRs) lack mCW, but mCW is enriched in mammalian interspersed repetitive (MIR) elements and LINE-2 elements. Both primate Alu and murine B1 elements are thought to be derived from 7SL-RNA^37^, and their evolutionary histories are independent^30^. In the brain, the Alu and B1 elements consistently lack mCW. In ESCs and iPSCs, mCW is significantly deficient in LINEs and LTRs, whereas mCW is significantly enriched in human Alu (Supplementary Fig. S7a) and mouse B1 (Supplementary Fig. S7b). In PGCs, mCW is mainly enriched in simple repeat regions (Supplementary Figs S7c,d, and S11). In oocytes of both species, mCW is consistently deficient in LTRs and MIRs but enriched in SINE elements (Supplementary Fig. S7e,f). The results in human oocytes were not significant, probably as a result of the limited sample numbers for analysis.

We also investigated gene-related regions. The results showed that gene bodies of ESCs and oocytes are enriched in mCW, whereas in the brain, mCW is depleted in the gene body (Fig. 3c,d; Supplementary Figs S8 and S9); these results were consistent between the two species. mCW and mCG are concordant across gene bodies in brain neurons, ESCs and oocytes. However, in PGCs, mCG is lower in the promoter than in the gene bodies (Supplementary Fig. S9), and the distribution of mCW is almost flat across the gene bodies and flanking regions (Supplementary Fig. S10). Our results present a general picture that enrichments of mCW in genomic regions are cell-type specific, and are also conserved.

### Young LINE-1 elements prefer low mCW levels in brain neurons

**Figure 4:**
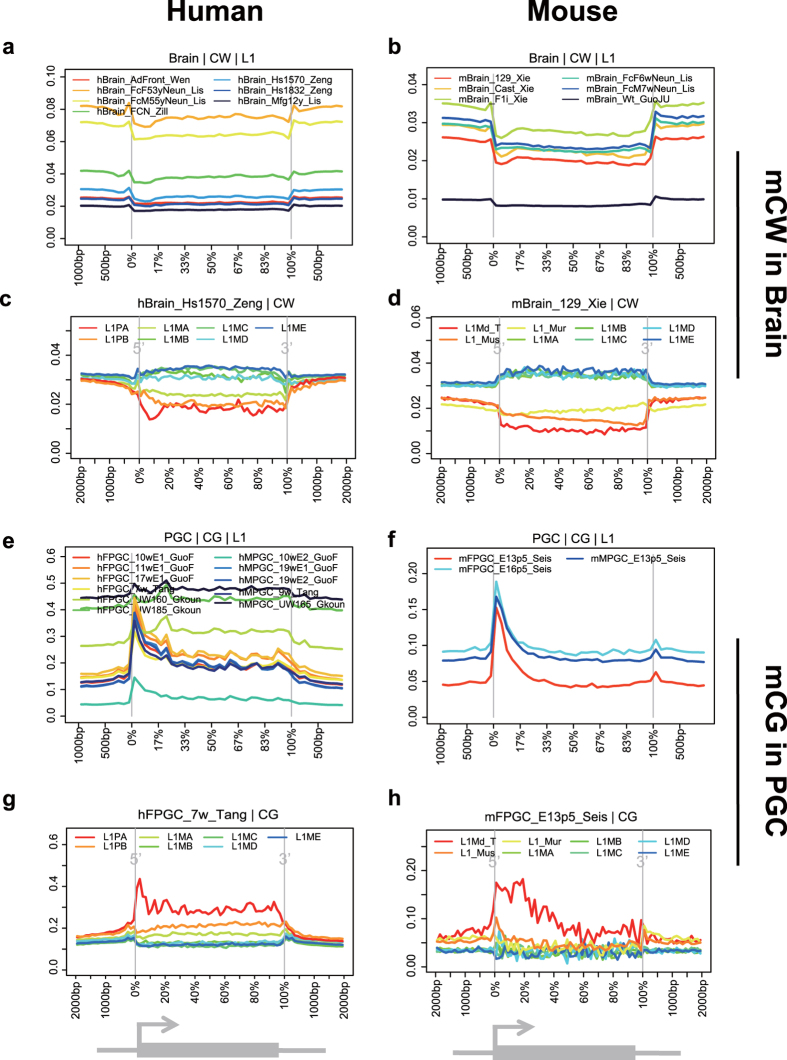
Young LINE-1 elements prefer lower mCW in brain and higher mCG at promoters in PGCs. Profiles of brain mCW across LINE-1 elements in humans (**a**) and mice (**b**). Examples of mCW profiles across subtypes of LINE-1 elements, including L1PA (youngest), L1PB, L1MA, L1MB, L1MC, L1MD, and L1ME (most ancient) in humans (**c**) and L1Md_T (youngest), L1_Mus, L1_Mur, L1MA, L1MB, L1MC, L1MD, and L1ME (most ancient) in mice (**d**), showing that young LINE-1 elements prefer lower mCW. Profiles of PGC mCG across LINE-1 elements in humans (**e**) and mice (**f**). Examples of mCG profiles across subtypes of LINE-1, from young to ancient in humans (**g**) and mice (**h**), showing that young LINE-1 elements prefer higher mCG at promoters.

We generated DNA methylation profiles across genes and transposons. Consistent with the enrichment analysis, all brain samples showed that the LINE-1 elements are devoid of mCW (Fig. 4a,b), but not mCG (Supplementary Fig. S14). It is known that LINE-1 elements constitute approximate 20% of the mammalian genome and are regulated by methyl-CpG-binding protein 2 (*MeCP2*)^38^. Additionally, mCH in neurons can be bound by *MeCP2*^32^. Our results indicate that LINE-1 activities in the brain may be regulated by mCW.

We further investigated methylation distribution within the sub-groups of LINE-1 elements. In humans, L1ME (most ancient), L1MD, L1MC, L1MB, L1MA, L1PB and L1PA (youngest) were investigated. Interestingly, the younger sub-groups prefer lower mCW levels (Fig. 4c, Supplementary Fig. S12). The ancient LINE-1 elements (from L1ME to L1MB) demonstrated higher mCW levels in the transcription region than in the flanking region. In mice, L1ME (most ancient), L1MD, L1MC, L1MB, L1MA, L1_Mur, L1_Mus and L1Md_T (youngest) were studied, and a similar phenomenon (Fig. 4d, Supplementary Fig. S12) was observed. Additionally, in mice, oocytes and two oocyte-similar MPGC samples lack mCW in LINE-1 elements, especially in younger LINE-1 elements (Supplementary Fig. S12). In additional to pre-existing knowledge that LINE-1 RNA is abundant in neurons^39^, our results indicated that LINE-1 elements are under the regulation of mCW, rather than mCG.

### Promoters of young LINE-1 elements prefer high mCG levels in PGCs

We also profiled the distributions of classical mCG in LINE-1 elements. Interestingly, both humans and mice showed a peak in mCG at the promoters of LINE-1 elements in PGCs (Fig. 4e,f), but not in other cell types (Supplementary Fig. S14). Specifically, the youngest LINE-1 sub-groups, human L1PA and mouse L1Md_T, showed the highest mCG levels in each PGC sample (Fig. 4g,h, Supplementary Fig. S13). At the PGC stage, CG dinucleotides are known to be globally demethylated^18^, and Tang *et al*. showed that younger LINE-1 elements tend to be less active^19^. Our results implied that, the young LINE-1 elements in PGCs are suppressed by the high mCG in the promoter region.

### Loci in human Alu and murine B1 elements prefer higher mCW levels during evolution

**Figure 5:**
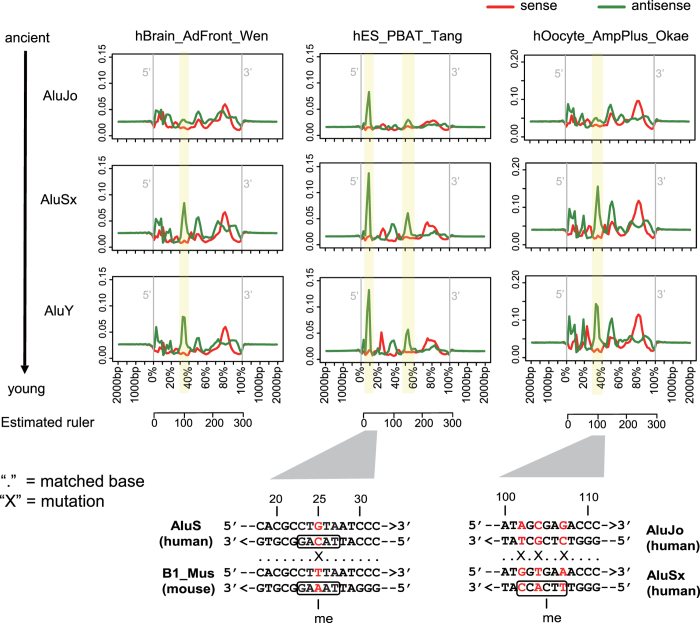
Young human Alu elements prefer mCW. Profiles of mCW across the sub-groups of human Alu elements from 500 bp upstream to 500 bp downstream in brain neuron, ES and oocyte. The mCW levels are shown for the sense strand (red) and antisense strand (green) separately. AluJo is the most ancient, and AluY is the youngest Alu elements. Yellow blocks mark the loci of Alu elements, where mCW levels increase with the evolution of Alu elements. The corresponding sequence mutations are shown below the profiles, and the nucleotides at the mutated positions are marked in red.

Alu elements have an important role in shaping the primate genome, and their retrotransposition rates are ten times higher than those of LINE-1 elements^29^. We observed several loci of human Alu elements that preferred higher mCW levels throughout evolution. Three main Alu sub-groups, including AluJo (ancient), AluSx and AluY (young), were studied. The mCW profile across the Alu elements showed a cell-type specific signature (Fig. 5). In ESCs/iPSCs, the highest peak of mCW was observed at the 5′ ends of Alu elements, which is consistent with our previous finding^28^. Interestingly, in human ESCs/iPSCs, we found an increase in mCW levels at the 5′ end peak position from the ancient AluJo to the younger AluSx (Fig. 5). In brain neuron and oocyte samples, we also found loci showing increased mCW levels from ancient to young Alu elements. Upon a closer investigation, we found that the antisense sequence from 102 bp to 107  bp is CT^m^CGCT in AluJo, and was mutated to TT^m^CACC in AluSx by transitions at three nucleotides, becoming a brain neuron and oocyte preferred mCH context, TN^m^CACC (Fig. 5).

B1 elements are the largest short interspersed nuclear element (SINE) family in rodents, with lengths of approximately 150 bp^30^. Similar to humans, we also found several loci in murine B1 elements with increased mCW levels during evolution (Supplementary Fig. S15). However, the mCW peak at the 5′ end of the B1 element is not as high as in human Alus (Fig. 5, Supplementary Fig. S15). Based on the conserved sequence backbone, the 5-mer context at 25 bp from the 5′ end of the B1 element in the TAAAG context, which is one nucleotide different from the corresponding cytosine site in the TACAG context in human Alu (Fig. 5). This different nucleotide may partially explain mouse ES does not prefer TA^m^CAG as does the human ES and as it may be under selection pressure of SINE elements.

### Strand-specific mCW is cell-type specific and is conserved among species

**Figure 6:**
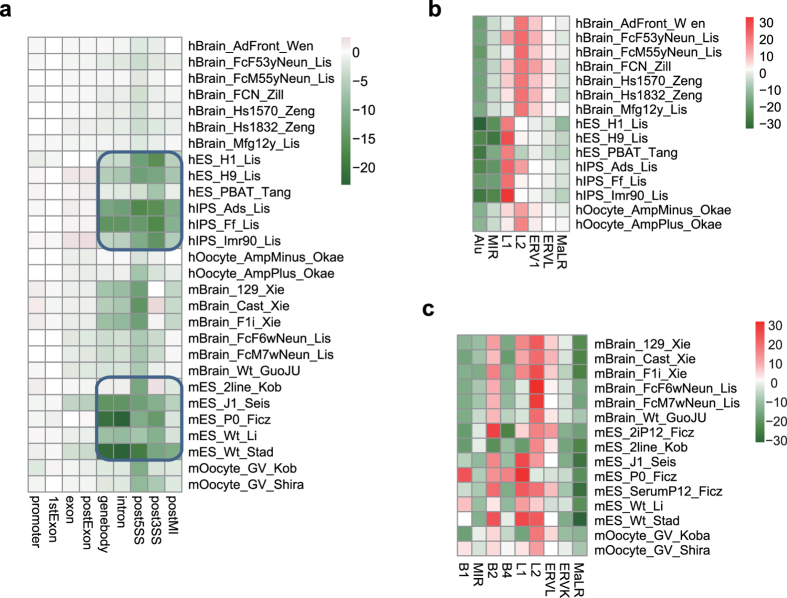
Strand-specific mCW in genes and TEs. (**a**) Heatmap for skewness of mCW in gene related regions, showing that intronic mCW is significant in both human and mouse ES samples. The colour index indicates the signed log_2_ *p* value, by two-tailed *t* test. Negative value, higher mCW on antisense strand; and *vice versa*. (**b,c**) Heatmaps for skewness of mCW in multiple transposon regions, including SINEs, LINEs and LTRs, in both humans (**b**) and mice (**c**).

Our previous work reported that mCH in human pluripotent cells is strand-skewed in introns and in SINE and LINE elements^28^. We then characterized the strand-specific distribution of mCW in gene-related regions and TEs. Human and murine ES/iPS samples also showed significantly higher mCW in the antisense strands in all of the intronic regions (Fig. 6a). The higher mCW in the sense strands of LINE-1 elements in ESC and LINE-2 elements in the oocyte and brain neuron samples are also consistent between the two species (Fig. 6b). Human Alu elements showed higher mCW in the antisense strand in all human samples. MIR showed higher antisense-strand mCW in most samples. Although the biological meaning of the strand-skewed mCW is unclear, our results indicate that the skewed distributions of mCW in SINEs and LINEs are conserved, and are also cell-type specific.

## Discussion

Previous studies of mammalian non-CG methylations either considered CH contexts as a whole^12^, or separated CH into CHG and CHH^11^. We developed new method, MiDD, and reported that CW and CC were the most significantly independent sub-contexts of mammalian mCH (Fig. 1). We also found that mCW was the representative context for non-CpG methylation.

Carrying out a two-dimensional comparison across both cell types and species, we found the sequence preference of mCW is cell-type specific and is also conserved between humans and mice (Fig. 2). Our subsequent enrichment studies of recurrent mCW in genes and repeat elements also confirmed this conclusion (Fig. 3, Supplementary Figs S7 and S8), and led to many novel findings. First, we found that the LINE-1 elements in brain neurons preferred lower mCW (Fig. 4a,b), indicating a regulation mechanism dependence on mCW rather than mCG. Second, younger LINE-1 elements in brain neurons preferred lower mCW in both humans and mice (Fig. 4c,d), suggesting a conserved roles for mCW in the evolution of LINE-1 elements. Third, we found several loci in Alu and B1 elements retaining higher mCW during evolution (Fig. 5 and 19), and we also found a local region with mCW-prone mutations from ancient AluJo to younger AluSx elements (Fig. 5). Fourth, in genes, we also found that mCW in ES intronic regions had the most significant skewness (Fig. 6). Fifth, mCW in mouse E16.5 MPGCs has an oocyte-like motif and distribution (Fig. 2, Supplementary Figs S9 and S16), which was confirmed by data from different laboratories. Taken together with the previous knowledge of increased mCG in mouse E16.5 MPGCs compared with E13.5 MPGCs^21^, the epigenetic signature suggested that late MPGCs may undergo an epigenetic regulation process similar to that of maturing female oocytes. Finally, beyond mCW, we also found that promoters of young LINE-1 elements in PGCs retained high mCG (Fig. 4e–h, Supplementary Fig. S13), whereas CG is globally demethylated in this stage. Until recently, the functions of mammalian non-CG methylations are largely unclear, many of our findings suggest that mCW may guide the evolution of TEs.

Our sequence preference analysis showed that brain and oocyte samples share a similar mCW motif, TN^m^CAC (Fig. 2). However, the distributions of mCW across chromosomes are largely different (Supplementary Fig. S4). Additionally, MIR elements showed significant enrichment of mCW in brain neuron samples (Fig. 3a,b) but were significantly deficient of mCW in oocytes (Supplementary Fig. S7f). Together, these results indicate that sequence preference can not fully explain the distributions of mCW.

We showed that the mCW motif is TA^m^CAG in human ES/iPS samples, and ^m^CA in most mouse ES samples. Although the mCW motifs of the mouse ES samples from *Smith et al*.^33^ are TA^m^CAG, the results lacks confirmation from independent work. One possibility is that the mCW motif is dynamically regulated during embryological development. It would be interesting to profile the DNA methylomes of different cell types in the early embryo. Our finding that the 5′ ends of B1 elements lost the TACAG pattern due to a C to T mutation involving human Alu elements, suggests that there may be weaker selection pressure on the TA^m^CAG motif in mouse ESCs.

In our cohort, the number of oocyte methylomes was limited, especially for humans. In the future, a larger number of samples would increase the statistical power. The recurrence phenomenon involving different samples and different species provides us with advantages to discriminate batch effects and inspect recurring mCW characteristics.

For mCC, we did not find any regular pattern as for mCW. Majority samples in our cohort have very low mCC levels (around 0.01), with a few exceptions (Supplementary Fig. S1b). The mCC-high samples are not specific in cell-type, in library type, or in species. We did not found any conserved sequence preference other than the CC dinucleotides (Fig. 1d, Supplementary Fig. S3). And the distribution of mCC across chromosomes are almost flat for most samples (Fig. 1e, Supplementary Fig. S4). It is still unclear whether the mCC is biological significant. But it is wise to discard mCC, and use mCW as the representative non-CG methylation pattern.

Our results demonstrate many highly conserved properties of mCW in humans and mice (Supplementary Table S2), and as well as evidence of cell-type specific distributions. We still have a limited understanding of mammalian non-CG methylations. There are far more cell types and species whose non-CG methylations remain to be explored. In the future, we hope to draw a full picture of mammalian non-CG methylations with DNA methylomes with more cell types and species.

## Methods

### Rebuilding DNA methylomes

DNA methylomes were downloaded from multiple resources (DRA000484, DRA000570, DRA000607, DRA003802, ERP001953, GSE11034, GSE16256, GSE30199, GSE30206, GSE34864, GSE37202, GSE42923, GSE46644, GSE46710, GSE49828, GSE51239, GSE52331, GSE56650, GSE61457, GSE63394, GSE63394, GSE63818, SRP057098) (Supplementary Table S1). Human methylomes were prepared based on hg18, and mouse methylomes were prepared based on mm9. The majority of the DNA methylomes were generated by realignment with raw sequences using BS-Seeker2^31^. We used Bowtie as the base aligner, trimmed the adapters, allowed up to 4% mismatches for one read and selected uniquely aligned reads for methylation calling. To avoid biased context calls proximal to mutated sites, we called single nucleotide variants (SNVs) from the ATCGmap files in the same way as described in Luz *et al*.^40^ and discarded the cytosines within 1 bp distance from the SNVs.

### Minimum dependence decomposition (MiDD)

An alternative to the maximum dependence decomposition (MDD) method^41^, MiDD was developed to study the subgroups of a motif. To find the novel context partition of mCH, we applied MiDD to the 6-mer context, NN^m^CHNN. As shown in Fig. 1a, we enumerated all 6-mer bipartitions based on each position. For each bipartition, we performed a chi-squared test on the methylation frequencies (numbers of occurrences in 1000 cases) of the 6-mer lists in the two subgroups. For each sample, the partition with the most significant *p*-value was selected. We selected the bipartition reported as the top significant bipartition by at least half of the samples. Then, MiDD method was applied within each sub-group hierarchically, until no significant bipartition was found (*p*-value < 0.05). Finally, the hierarchical motif tree was constructed.

### Normalized motif logo based on 6-mers

Given that the frequencies of 6-mers (NN^m^CNNN) were unbalanced throughout the genome, we applied a normalization method for estimating the nucleotide frequencies at each position of the 6-mers. The 6-mers containing the CCGG sub-context were discarded so as to make RRBS and WGBS comparable on non-CG context. First, the average methylation levels of the 6-mer patterns were independently calculated throughout the genome, noted as *M*_*w*_, where *w* is one 6-mer. At each position (*p*) in the 6-mer, we calculated the weight of each nucleotide (*n*, *n*∈{*A*, *C*, *G*, T}) as


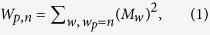


where *w*_*p*_ indicates the nucleotide at position *p* of the 6-mer pattern *w*. The normalized frequency of each nucleotide was calculated as


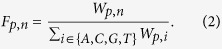


Logo plots were generated by WebLogo^42^.

### Enrichment study of recurrent methylated CW sites in specific regions

The recurrent mCW sites among multiple non-RRBS samples were prepared for each cell type. For the brain neuron, ESC/iPSC and PGC samples, the recurrent CW sites were defined as coverage ≥4 reads for at least 4 samples (background). Within all of the background sties, the recurrent methylated CW sites (foreground) were defined as the top 10% methylated mCW in each sample for at least 75% of the samples. Given the low number of oocyte samples in each species, we merged sites from multiple samples together as a single meta-methylome. The sites with the top 80% coverage were used as background. Within the background the top 10% methylated sites were used as foreground. Then, the fold enrichment of mCW in specific regions was measured by the log_2_ odd-ratios by comparing both foreground and background sites in the region.

### Strand-specific study of mCW

As shown in Fig. 6, we calculated the strand-specific distribution of mCW as previously described^28^. The signed log_2_ *p*-values are used to represent the significance of skewness. Positive numbers indicate higher methylation levels on the sense strand, and *vice versa*.

### Investigated genomic regions

The intergenic region is defined as the region in length of 1 k bp, which is 10 k bp upstream of transcription starting sites (TSS). The gene-related regions include the promoter (from 500 bp upstream of the TSS to 100 bp downstream of the TSS), 1st exon (1stExon), posterior (2nd and later) exon (postExon), posterior 5′SS region (post5SS), posterior MI (middle intron) region (postMI) and posterior 3′SS region (post3SS). The 5′SS, MI and 3′SS regions are defined as in our previous study^28^. The transposon regions include SINE, LINE, LTR and other DNA repeat families, annotated in the RepeatMasker dataset from UCSC (http://genome.ucsc.edu/).

## Additional Information

**How to cite this article**: Guo, W. *et al*. Mammalian non-CG methylations are conserved and cell-type specific and may have been involved in the evolution of transposon elements. *Sci. Rep*. **6**, 32207; doi: 10.1038/srep32207 (2016).

**Accession Code**: The DNA methylomes are available in the Gene Expression Omnibus repository [GSE77019].

## Supplementary Material

Supplementary Information

## Figures and Tables

**Table 1:** All the gathered DNA methylomes.

Human (26)				
Brain	ESC/iPSC*	FPGC	MPGC	Oocyte
7	6	5	5	3
AdFront_Wen	H1_Lis	10wE1_GuoF	10wE2_GuoF	AmpMinus_Okae
FcF53yNeun_Lis	H9_Lis	11wE1_GuoF	19wE1_GuoF	AmpPlus_Okae
FcM55yNeun_Lis	PBAT_Tang	17wE1_GuoF	19wE2_GuoF	*Rrbs_GuoHS*
FCN_Zill	Ads_Lis*	7w_Tang	9w_Tang	
Hs1570_Zeng	Ff_Lis*	UW160_Gkoun	UW165_Gkoun	
Hs1832_Zeng	Imr90_Lis*			
Mfg12y_Lis				
**Mouse (25)**				
**Brain**	**ESC**	**FPGC**	**MPGC**	**Oocyte**
**8**	**8**	**2**	**3**	**4**
129_Xie	2line_Kob	E13p5_Seis	E13p5_Seis	GV_Kob
Cast_Xie	J1_Seis	E16p5_Seis	E16p5_Kob	GV_Shira
F1i_Xie	*p01Rrbs_Smith*		E16p5_Seis	*Rrbs_GuoF*
FcF6wNeun_Lis	*p02Rrbs_Smith*			*Rrbs_Shen*
FcM7wNeun_Lis	*p032Rrbs_Smith*			
*Rrbs_Meis*	P0_Ficz			
*Rrbs_Smith*	Wt_Stad			
Wt_GuoJU	Wt_Li			

26 human methylomes and 25 murine methylomes were collected. Cell types include brain neurons (Brain), embryonic stem cell (ESC), induced pluripotent stem cell (iPSC), female PGC (FPGC), male PGC (MPGC), and oocyte. The iPS cell lines are marked with asterisk. Samples in RRBS libraries are marked in italic. In the main text, samples are named with a prefix using their cell types, such as *hBrain_FCN_Zill* for the human brain neuron sample FCN_Zill.
