# Erratum: Mammalian non-CG methylations are conserved and cell-type specific and may have been involved in the evolution of transposon elements

**DOI:** 10.1038/srep39440

**Published:** 2017-03-15

**Authors:** Weilong Guo, Michael Q. Zhang, Hong Wu

Scientific Reports 6: Article number: 3220710.1038/srep32207; published online: 08 30 2016; updated: 03 15 2017

This Article contains an error in Table 1. ‘Mfg12y_Lis’ should appear in a single column under the heading ‘Brain’. The correct Table 1 appears below.

## Figures and Tables

**Table 1:** 

Human (26)				
Brain	ESC/iPSC*	FPGC	MPGC	Oocyte
7	6	5	5	3
AdFront_Wen	H1_Lis	10wE1_GuoF	10wE2_GuoF	AmpMinus_Okae
FcF53yNeun_Lis	H9_Lis	11wE1_GuoF	19wE1_GuoF	AmpPlus_Okae
FcM55yNeun_Lis	PBAT_Tang	17wE1_GuoF	19wE2_GuoF	*Rrbs_GuoHS*
FCN_Zill	Ads_Lis*	7w_Tang	9w_Tang	
Hs1570_Zeng	Ff_Lis*	UW160_Gkoun	UW165_Gkoun	
Hs1832_Zeng	Imr90_Lis*			
Mfg12y_Lis				
**Mouse (25)**				
**Brain**	**ESC**	**FPGC**	**MPGC**	**Oocyte**
8	8	2	3	4
129_Xie	2line_Kob	E13p5_Seis	E13p5_Seis	GV_Kob
Cast_Xie	J1_Seis	E16p5_Seis	E16p5_Kob	GV_Shira
F1i_Xie	*p01Rrbs_Smith*		E16p5_Seis	*Rrbs_GuoF*
FcF6wNeun_Lis	*p02Rrbs_Smith*			*Rrbs_Shen*
FcM7wNeun_Lis	*p032Rrbs_Smith*			
*Rrbs_Meis*	P0_Ficz			
*Rrbs_Smith*	Wt_Stad			
Wt_GuoJU	Wt_Li			

