# Supplementary material for: Mammalian non-CG methylations are conserved and cell-type specific and may have been involved in the evolution of transposon elements
Source: Sci Rep. 2016 Aug 30;6:32207. doi: 10.1038/srep32207 (PMC5004121; doi:10.1038/srep32207)
Supplement: Supplementary Information [file srep32207-s1.pdf]

Mammalian non-CG methylations are conserved and cell-type specific and may have been involved in the evolution of transposon elements

Weilong Guo, Michael Zhang, Hong Wu

Supplementary Figures

Fig. S1-S16

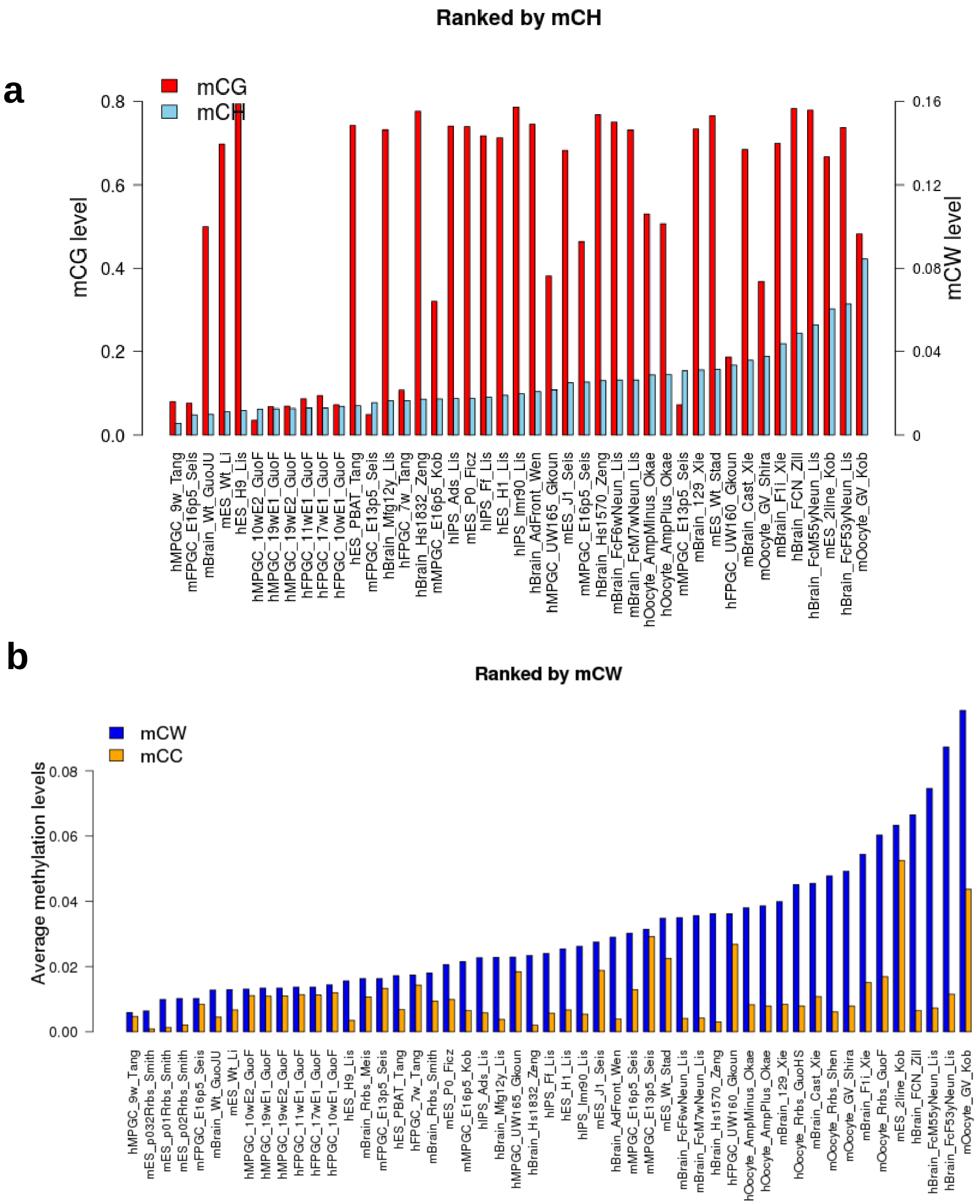

Supplementary Figure S1. Cross-sample study of the bulk methylation levels in different contexts. (a) The bulk methylation levels of CG and CH contexts (y-axis) are discordant across samples. Samples in RRBS library are excluded to avoid bias of CG methylation. (b) The bulk methylation levels in CW and CC contexts are discordant across all the samples.

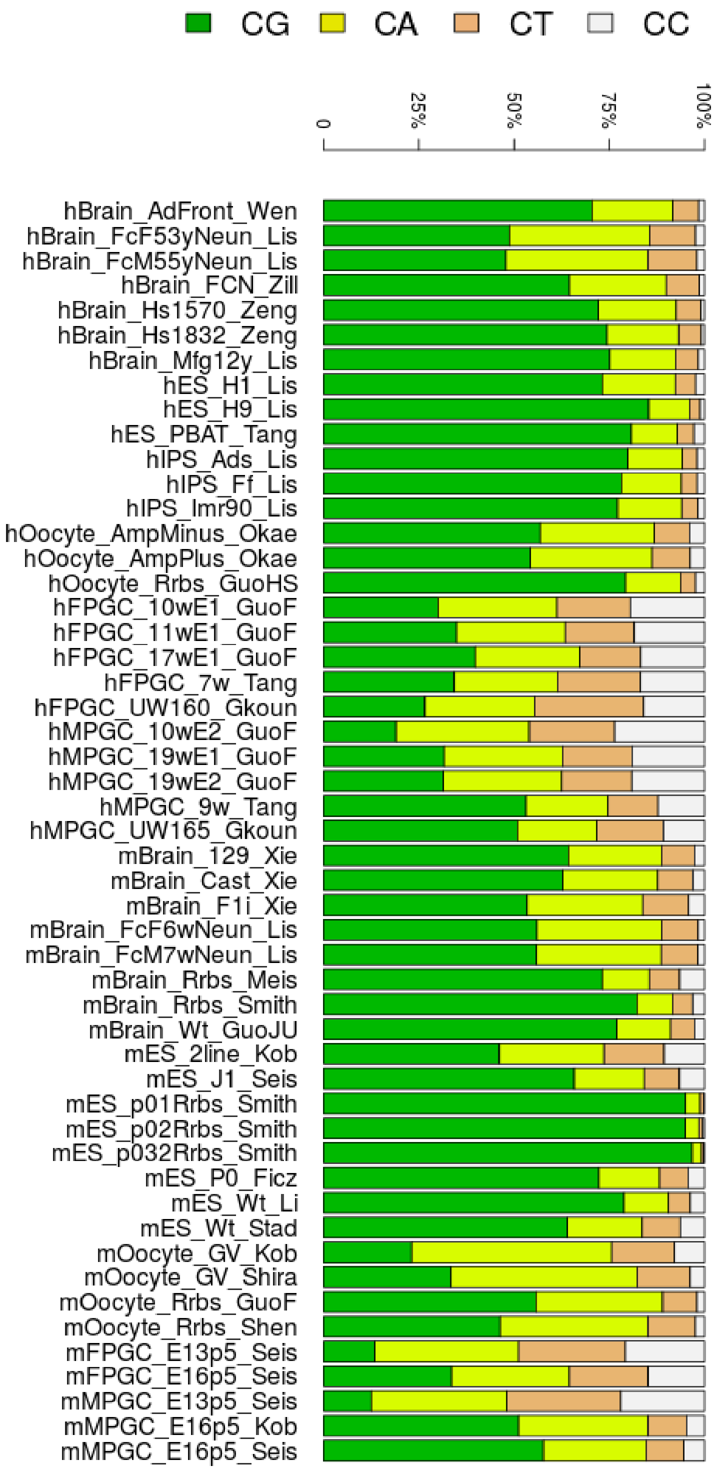

Supplementary Figure S2. The contributions to overall DNA methylations in CA, CC, CG and CT contexts. The percentage indicates how much chance belonging to each context if a cytosine is observed as methylated.

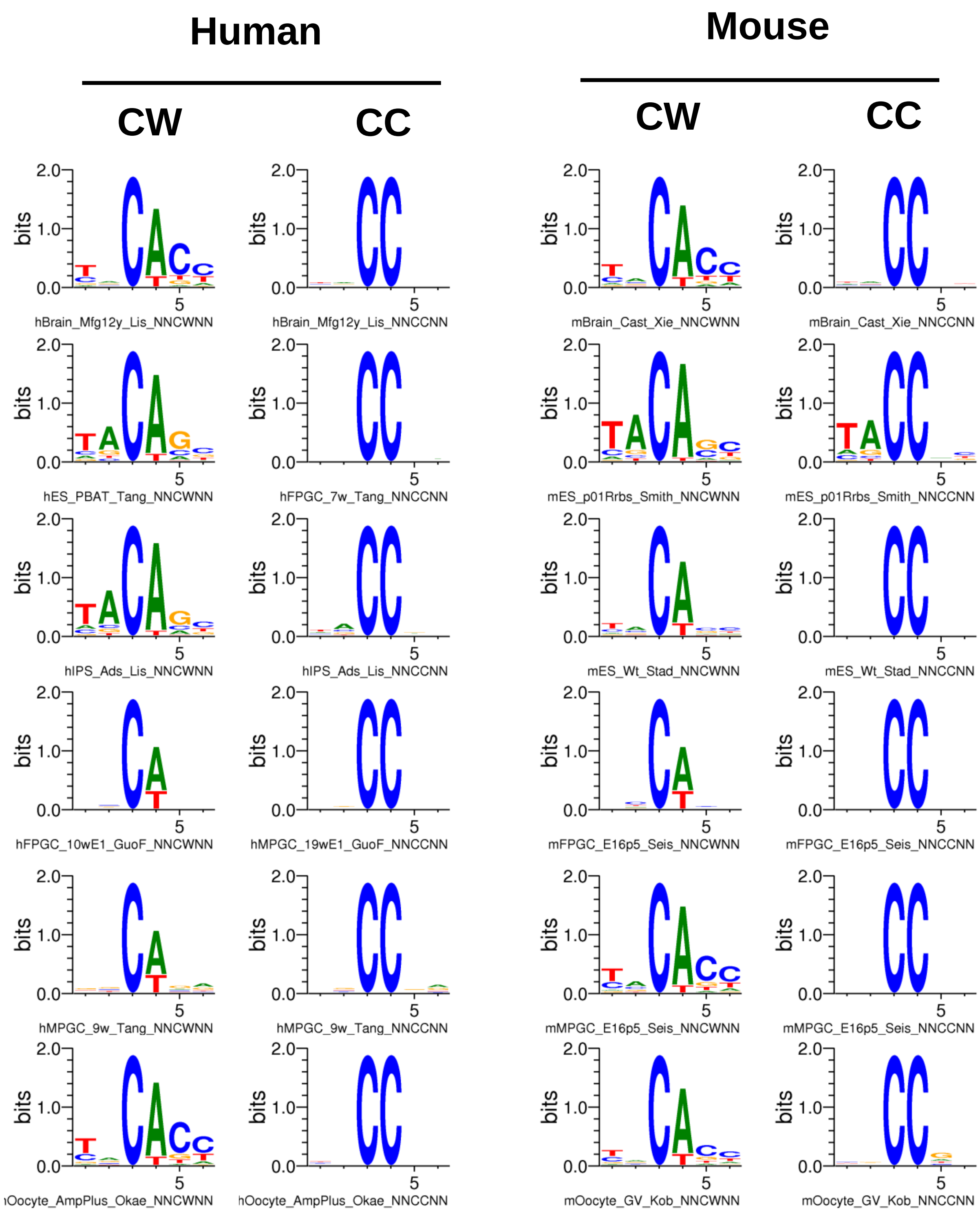

Supplementary Figure S3. The logos of normalized motifs for mCW and mCC in selected samples. Examples are selected as covering all cell types (brain, ES/iPS, MPGC, FPGC and oocyte) in both human and mouse. All samples showed that mCW and mCC are different in motifs.

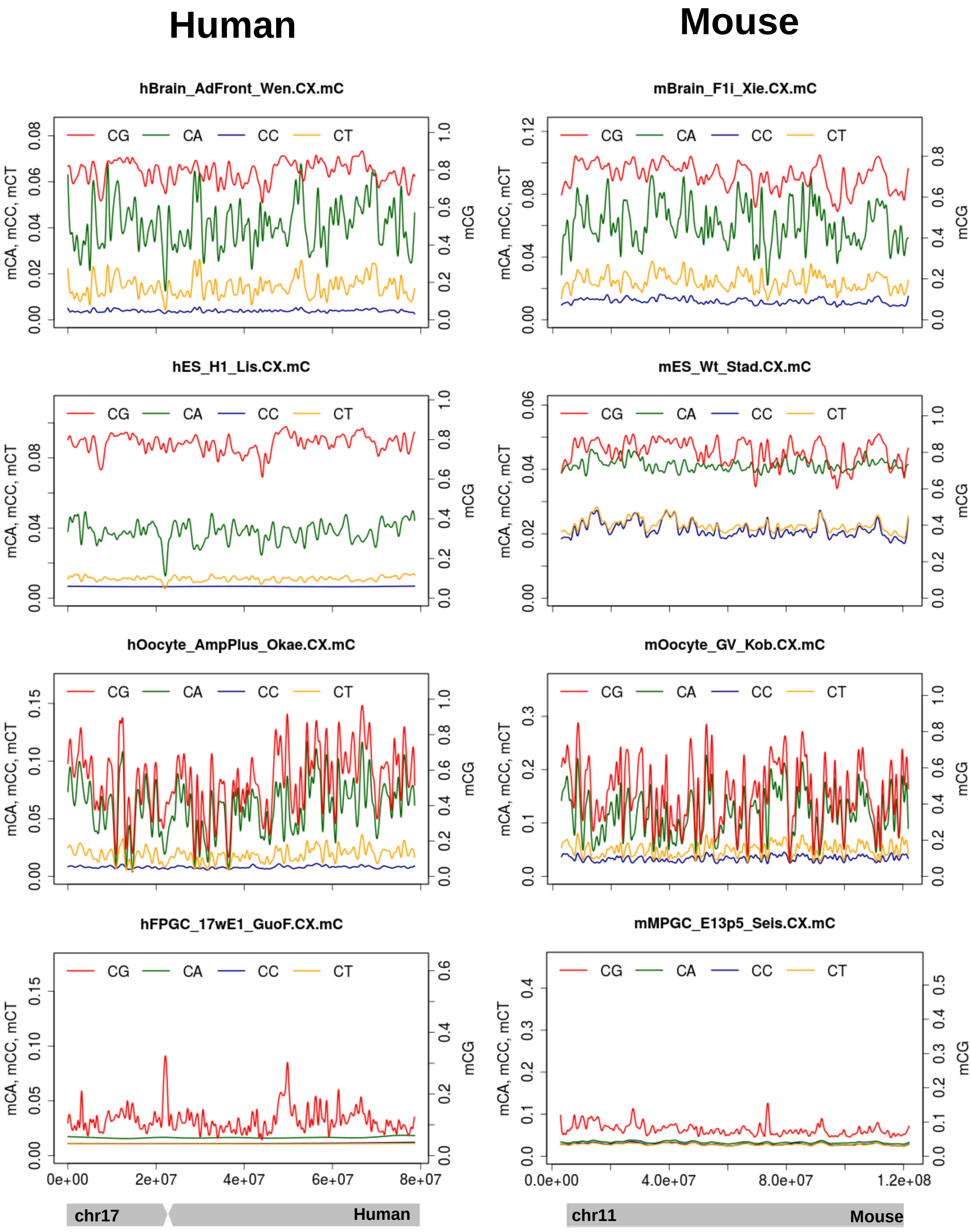

Supplementary Figure S4. The methylation level profiles in four contexts (CA, CC, CG and CT) across one chromosome. One example is selected for each cell type in both human and mouse. The average methylation levels are smoothed in bins (bin size, 20k bp).

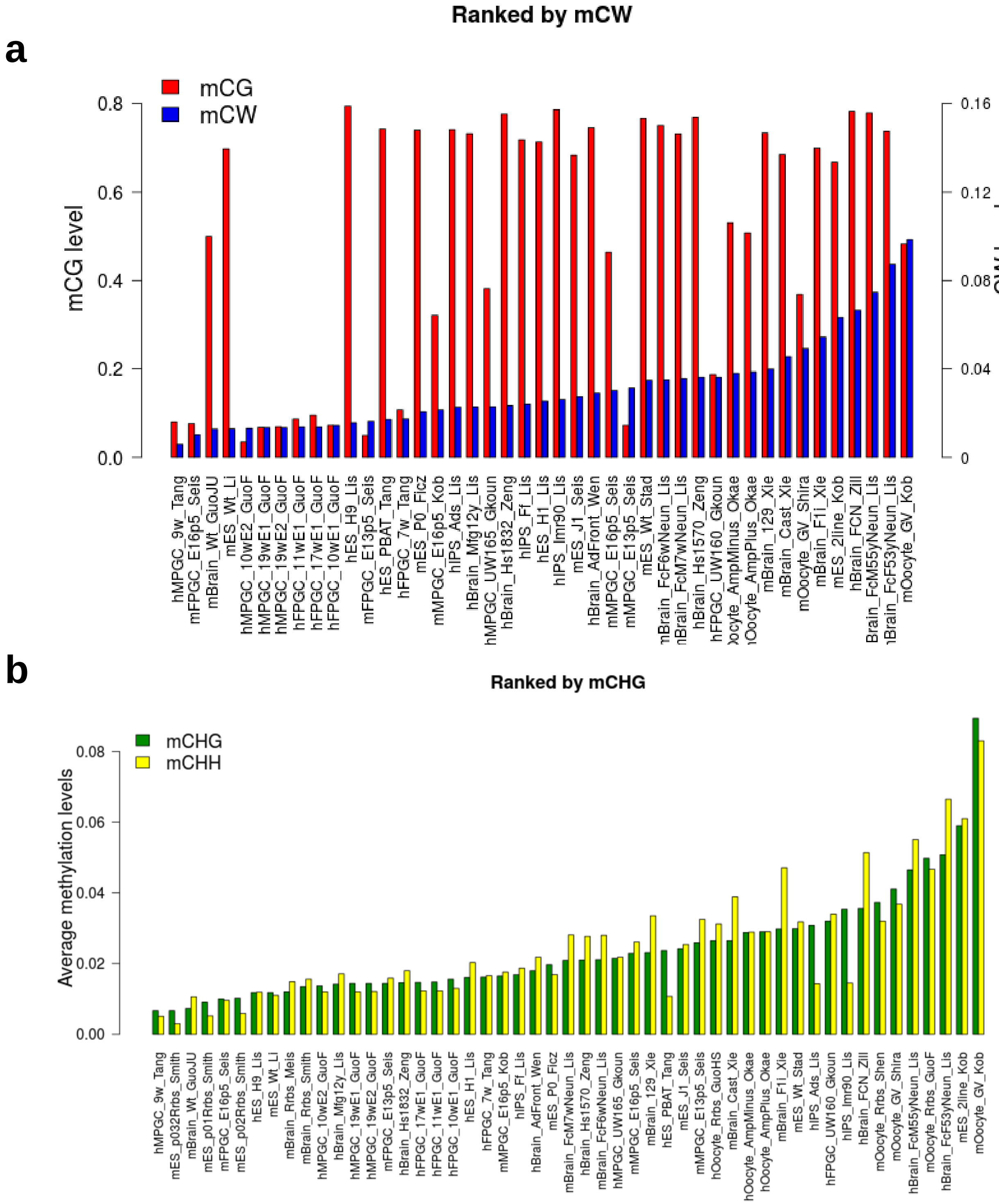

Supplementary Figure S5. Bulk methylation studies show (a) mCG and mCW are discordant, (b) mCHG and mCHH are concordant.

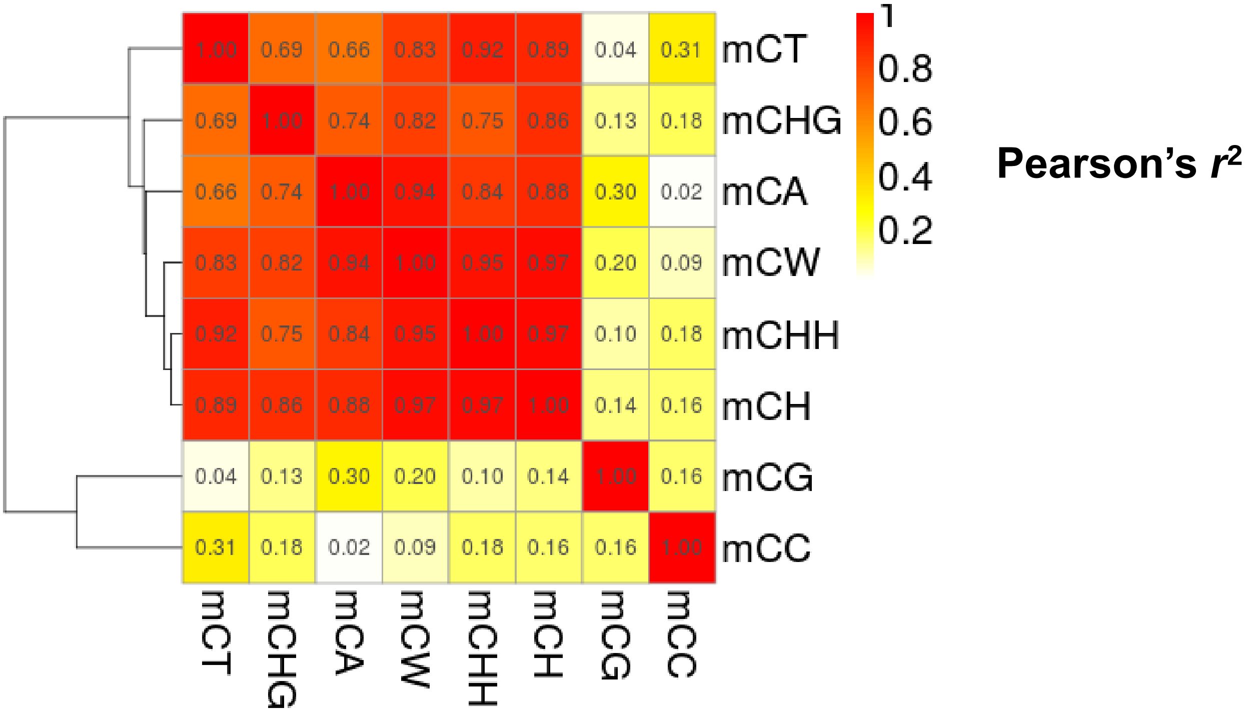

Supplementary Figure S6. Heatmap of the squared correlation coefficient. Pearson's r is calculated by the average methylation levels of each context pair across all samples. Distance for building hierarchical tree is used as $(1 - r^2)$. W = {A, T} and H = {A, C, T}. mCG and mCC are discordant with mCW. The mCHG and mCHH levels are highly correlated.

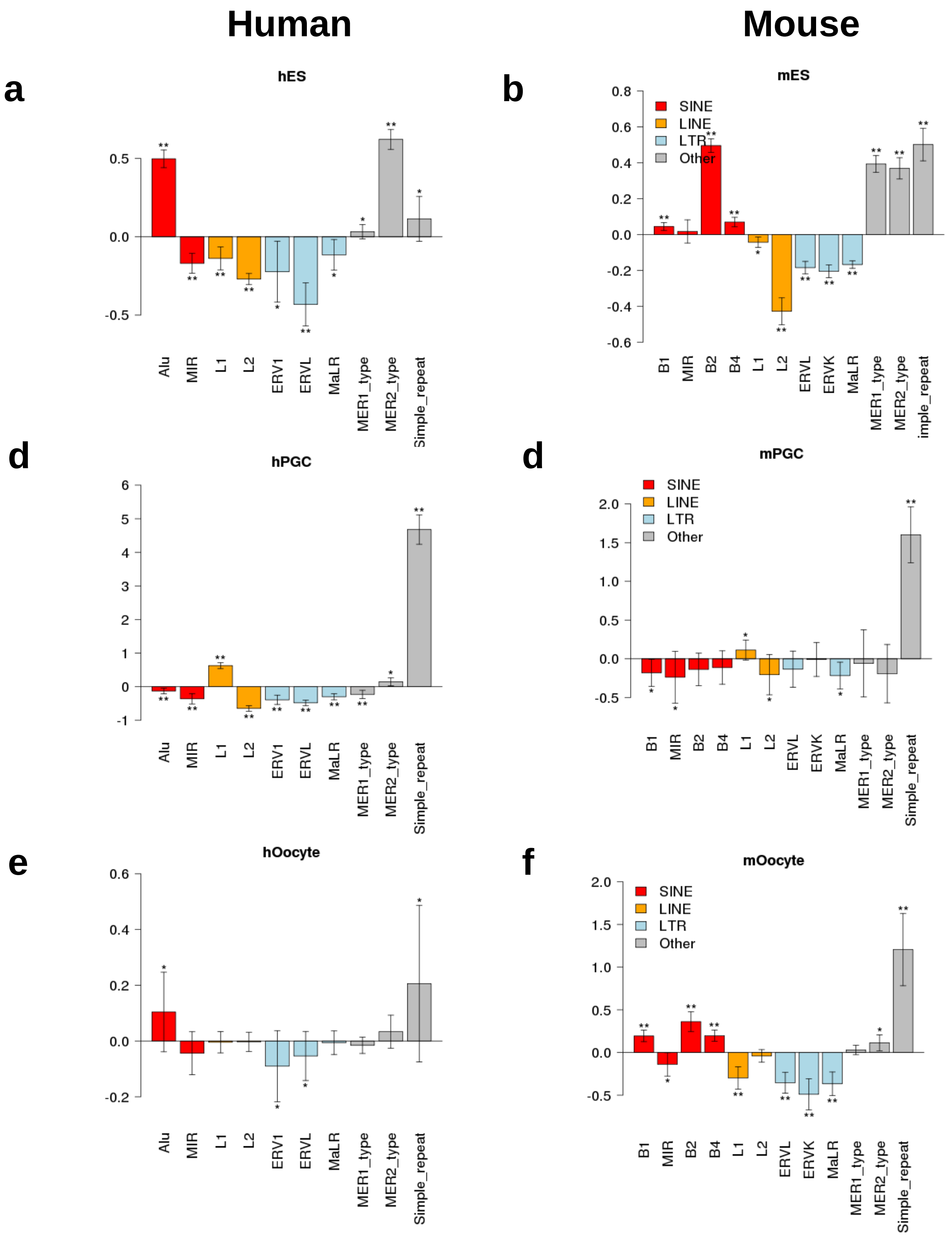

Supplementary Figure S7. Enrichment study of mCW in the repeat elements in ES/iPS (a, b), PGC (c, d) and oocyte (e, f). The repeat elements investigated include SINEs, LINEs, LTRs and other elements. Y-axis, enrichment score, defined as the $\log_2$ fold changes between observed high mCW site count and expected high mCW site count. The mean (bar height) and standard deviation (error bar) are calculated using enrichment scores of all the autosomes. The mCW-enriched elements in each cell type (ES, PGC, oocyte) are consistent between human (a, c, e) and mouse (b, d, f).

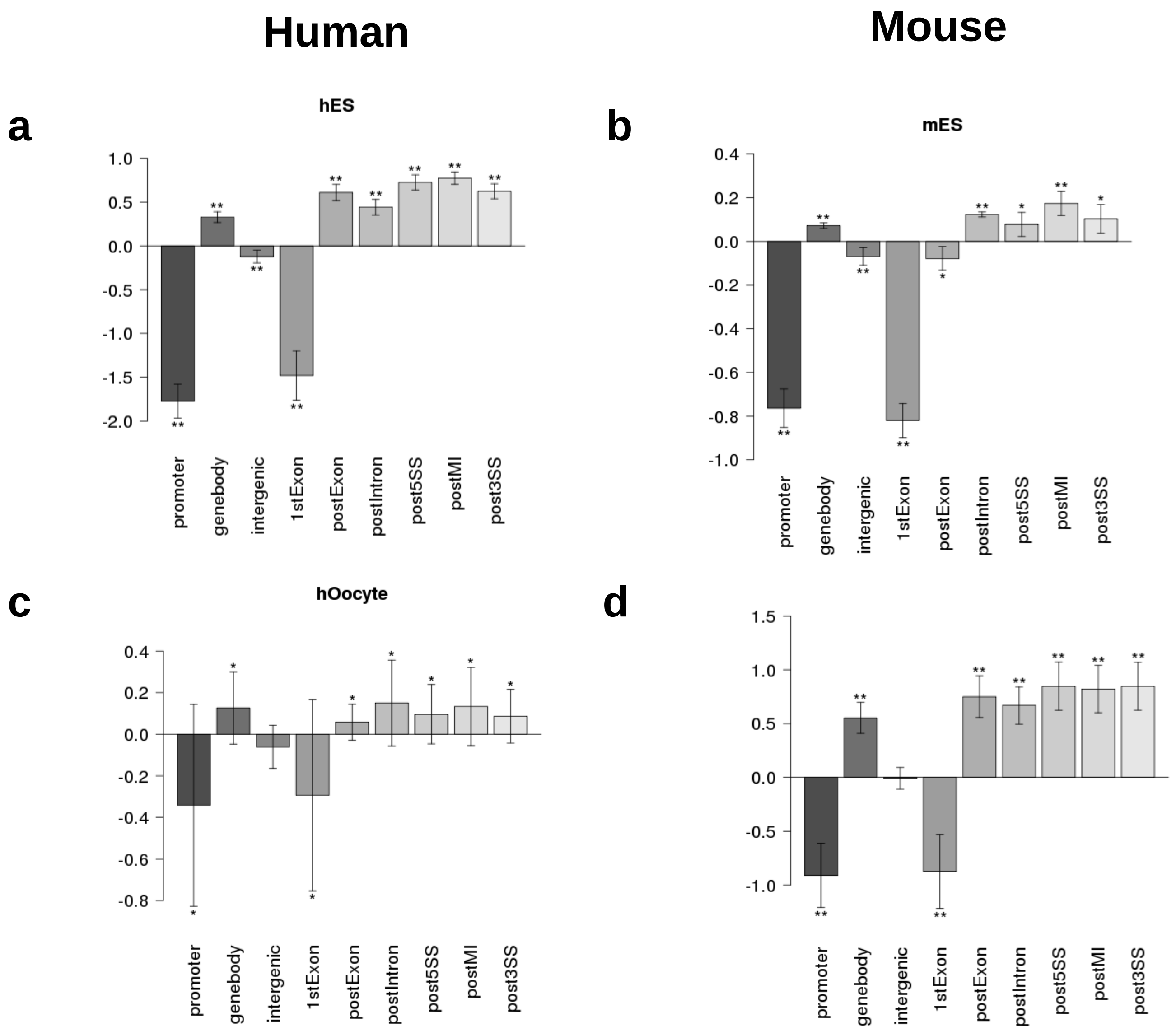

Supplementary Figure S8. Enrichment study of mCW in gene related region in ES/iPS (a, b) and PGC (c, d) The gene related regions are separated as promoter, gene body, intergenic regions, first exon (1stExon) and posterior exons (postExon), introns (postIntron), 5' Splicing Site region (post5SS), Middle Intron (postMI) and 3' Splicing Site region (post3SS). Y-axis, enrichment score, defined as the $\log_2$ fold changes between observed high mCW site count and expected high mCW site count. The mean (bar height) and standard deviation (error bar) are calculated using enrichment score from all the autosomes. The mCW-enriched regions in each cell type are consistent between human (a, c) and mouse (b, d).

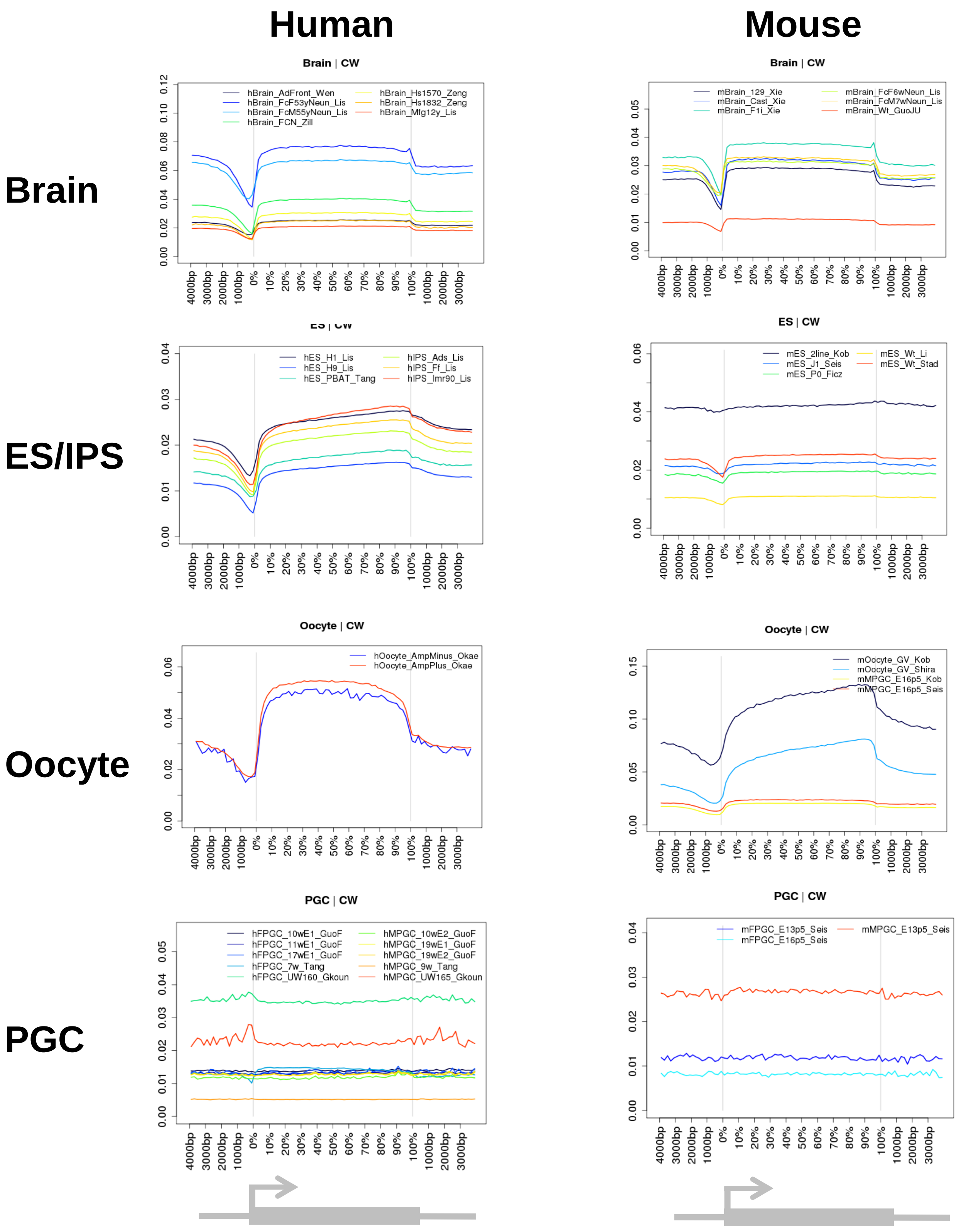

Supplementary Figure S9. Profiles of mCW across gene body regions in all cell types. The distributions of mCW from 4k bp upstream of transcription starting sites (TSS) to 4k bp downstream of transcription terminal sites (TTS). RRBS samples are excluded considering bias of mCG. The mouse MPGC samples in E16.5 are grouped with mouse oocyte samples.

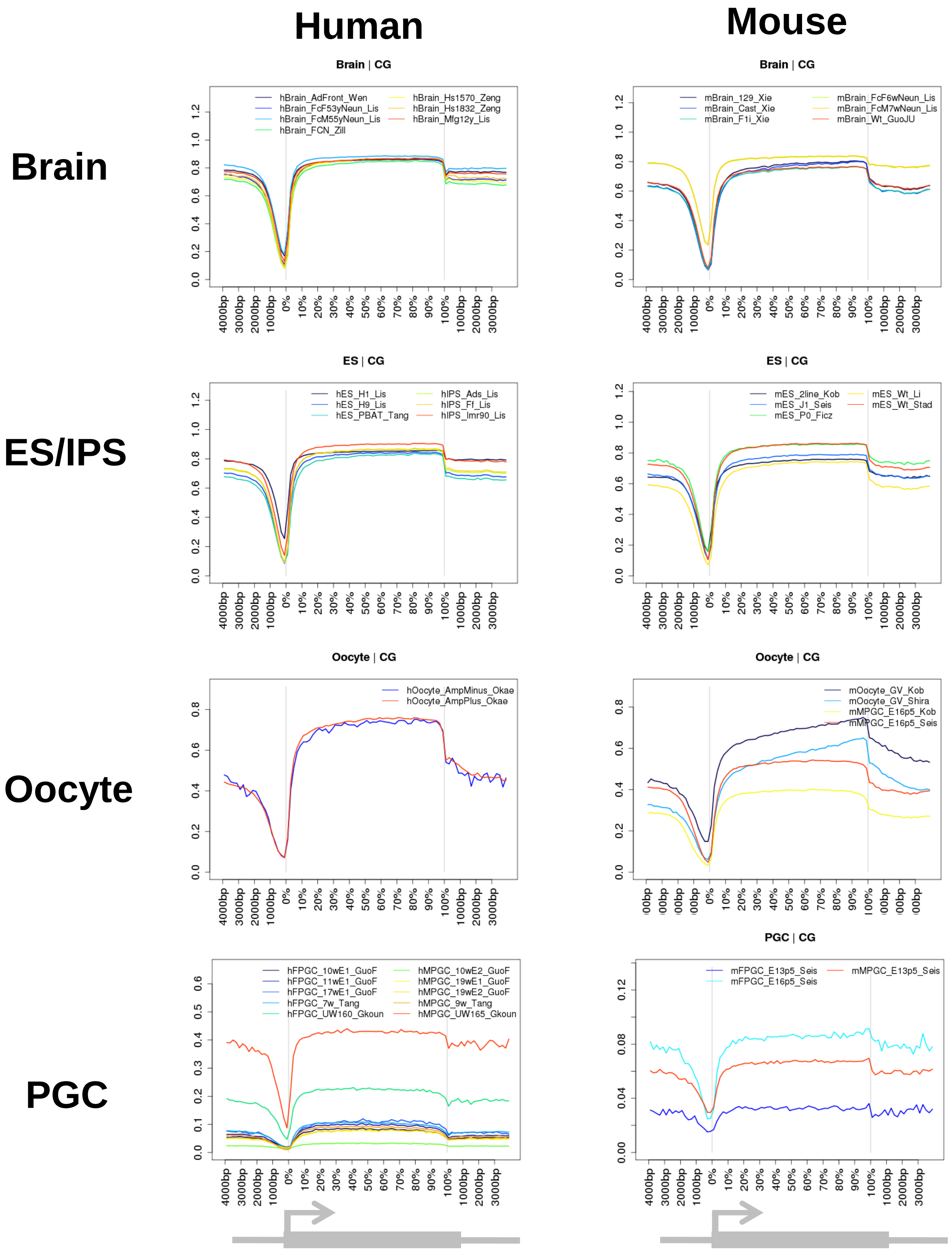

Supplementary Figure S10. Profiles of mCG across gene body regions. The distributions of mCG from 4k bp upstream of transcription starting sites (TSS) to 4k bp downstream of transcription terminal sites (TTS). RRBS samples are excluded considering bias of mCG. The mouse MPGC samples in E16.5 are grouped with mouse oocyte samples.

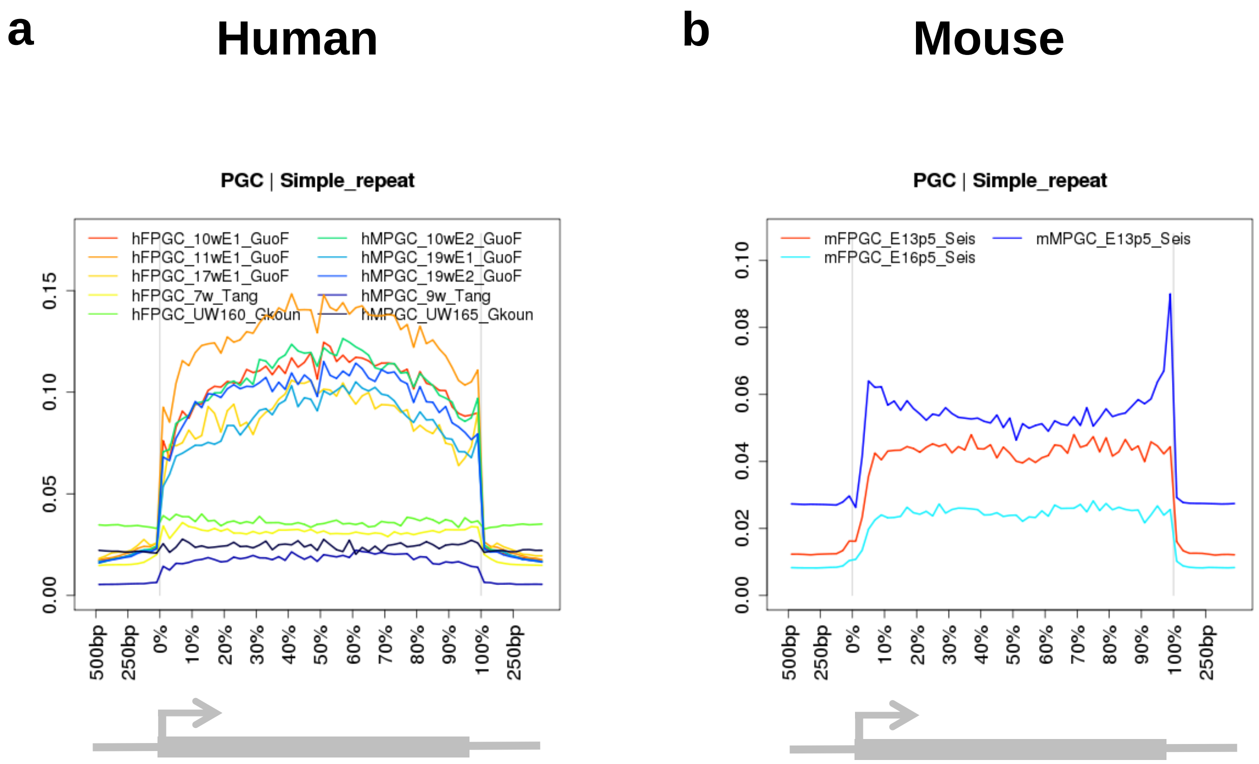

Supplementary Figure S11. Profiles of PGC mCW across simple repeats. The distributions of mCW across the simple repeat regions with 500 bp on each boundary in both human (a) and mouse (b).

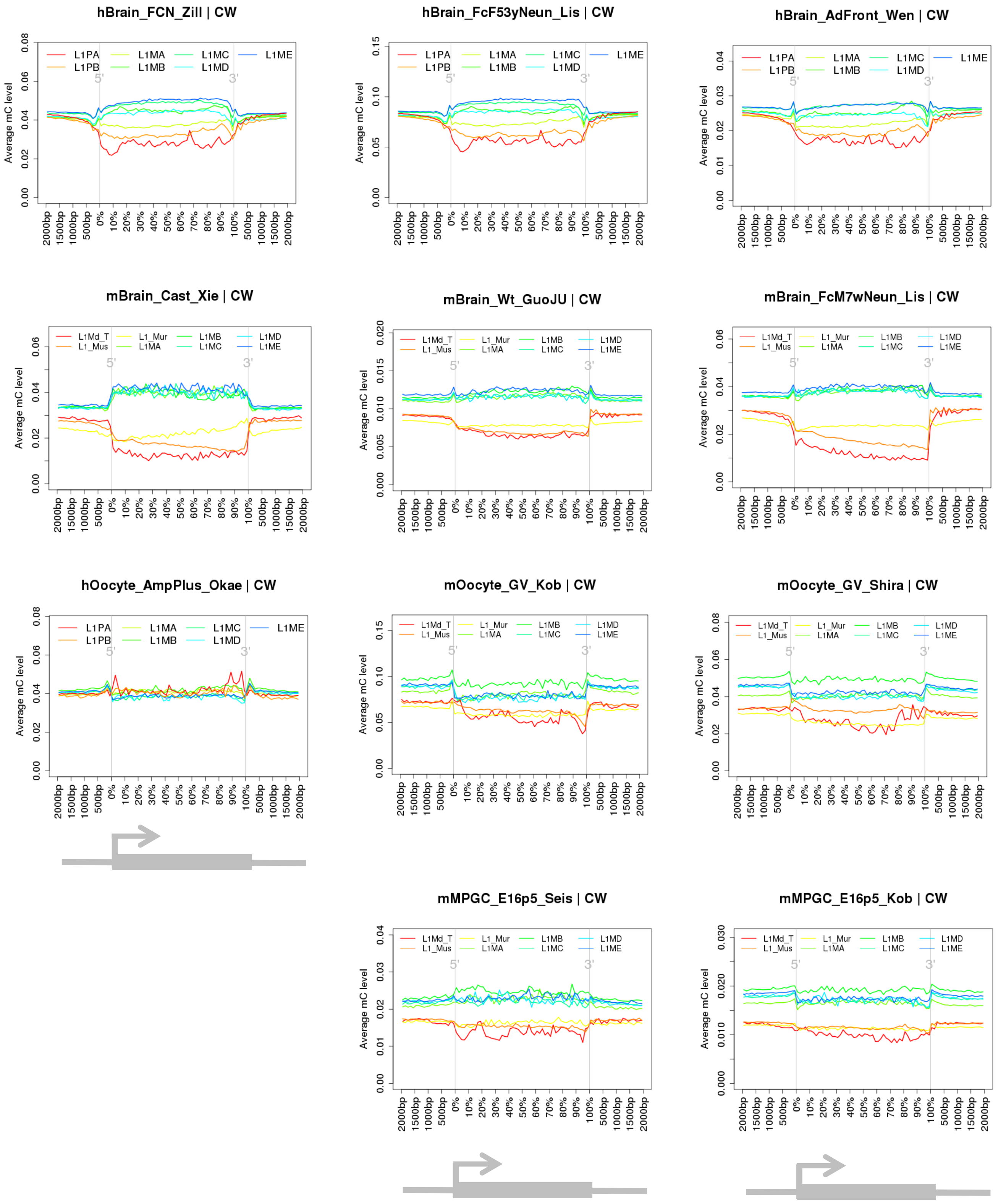

Supplementary Figure S12. Profiles for mCW across different sub-groups of LINE-1 elements. Examples of mCW profiles across subtypes of LINE-1 elements, including L1PA (youngest), L1PB, L1MA, L1MB, L1MC, L1MD, L1ME (most ancient). In all the brain neuron samples and mouse oocyte (-like) samples, younger LINE-1 elements prefer lower mCW.

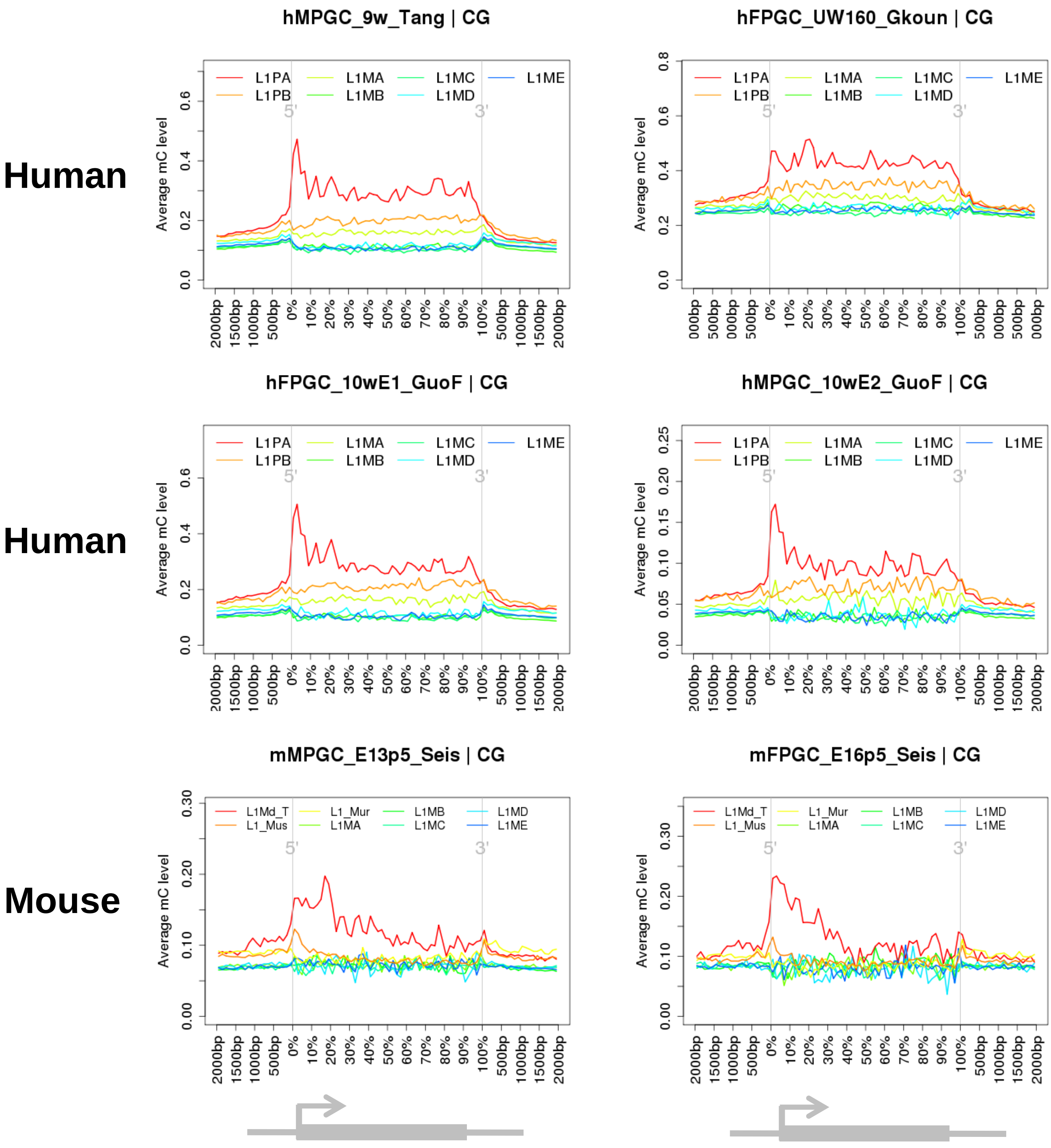

Supplementary Figure S13. Profiles for PGC mCG across different sub-groups of LINE-1 elements. Examples of mCG profiles across subtypes of LINE-1 elements, including L1PA (youngest), L1PB, L1MA, L1MB, L1MC, L1MD, L1ME (most ancient) in multiple examples. In PGCs, there are peaks of mCG at the promoters of young LINE-1 elements.

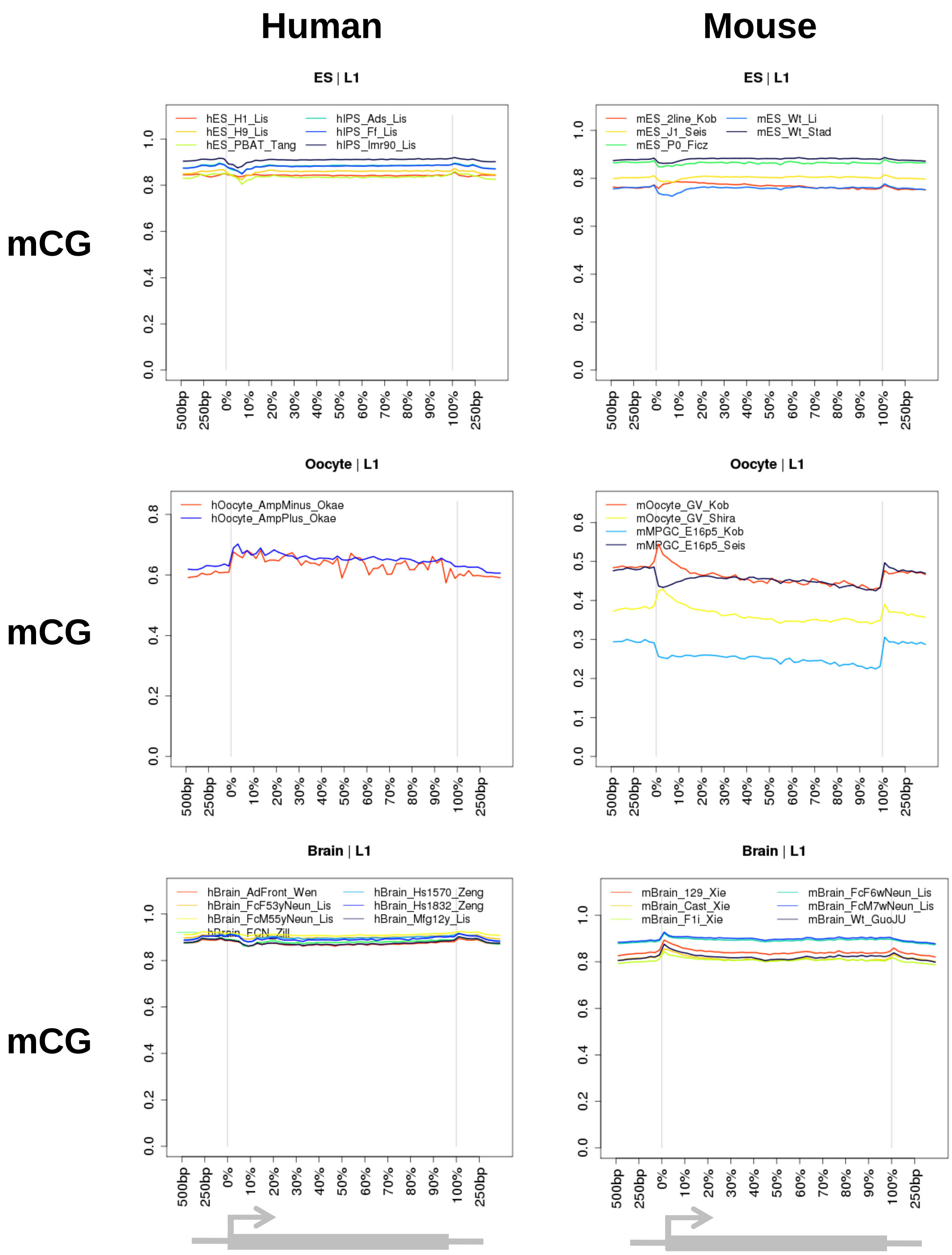

Supplementary Figure S14. Profiles of mCG across LINE-1 elements in ES/iPS, oocyte and brain neurons. The distributions of mCG across the LINE-1 elements with 500 bp at boundaries in both human and mouse. RRBS ware excluded. The mouse MPGC samples in E16.5 are grouped with mouse oocyte samples.

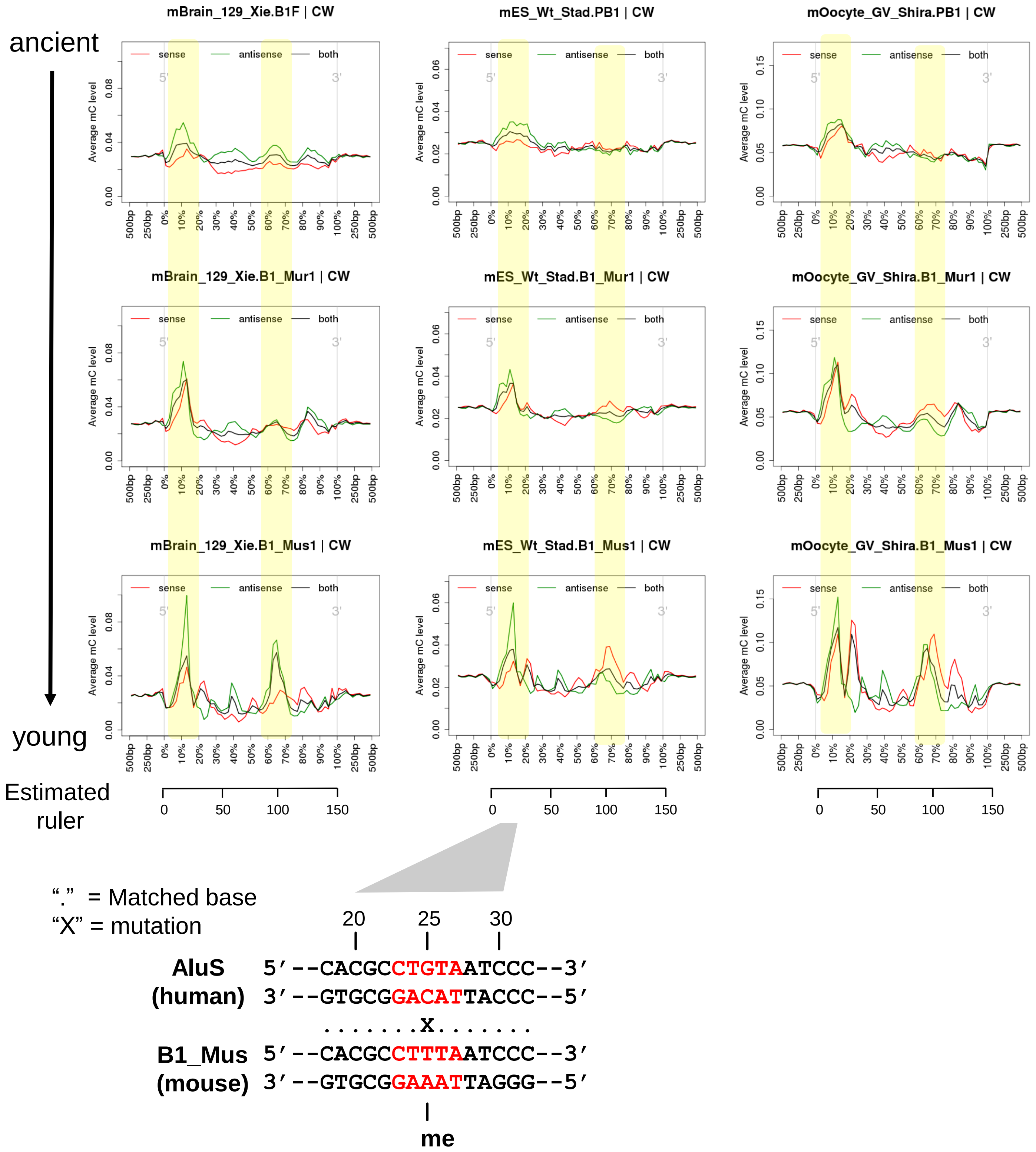

Supplementary Figure S15. Loci in young murine B1 elements prefer high mCW methylation. Profiles of mCW across the sub-groups of murine B1 elements from 500 bp upstream to 500 bp downstream in brain, ES and oocyte. The sub-groups of B1 elements, B1F, B1_Mur1 and B1_Mus1 are ranked from most ancient to youngest. Yellow blocks marked the loci of B1 elements, with increased mCW levels during evolution. The corresponding sequence mutation between murine B1 and human Alu were shown under the profiles.

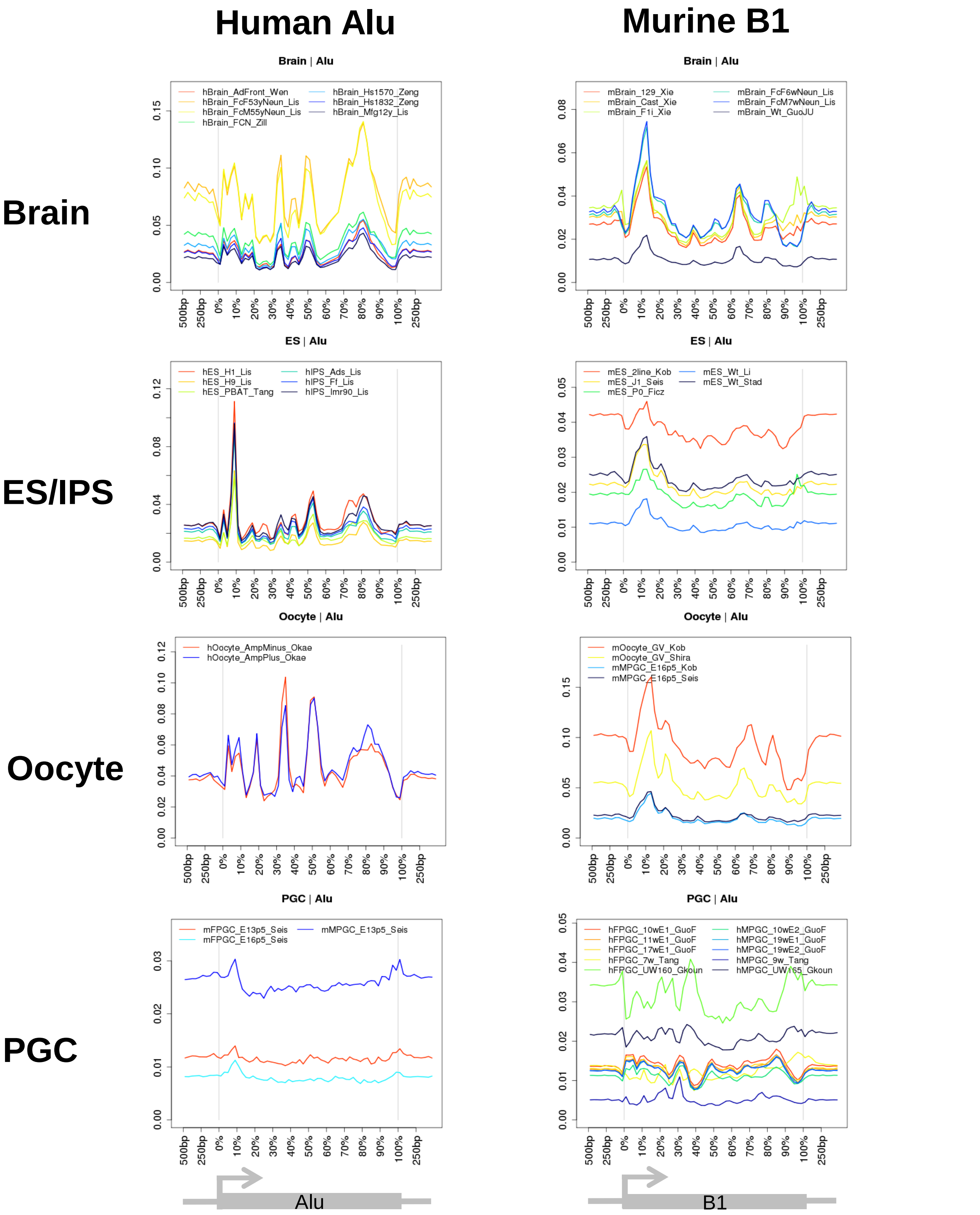

Supplementary Figure S16. Profiles of mCW across human Alu and murine B1 elements. The distributions of mCW across human Alu elements and murine B1 elements with 500bp at each boundary in all cell types. RRBS samples are excluded. The mouse MPGC samples in E16.5 are not include.

Supplementary Tables

Table S2

Supplementary Table S1. Sources of the DNA methylomes.
A table list for all detailed information of the DNA methylomes gathered in this study.

(Additional File 1, XLSX file)

Supplementary Table S2. Summary of the conserved and cell-type specific properties of mammalian mCW and mCG.

	NO.	Conserved between human and mouse	Specific in cell-type
mCH	1	mCW is abundant in ES, brain, oocyte and PGC	
	2	mCW and mCC are most significant independent non-CG contexts	
	3	Motifs of mCW are conserved	Motifs of mCW are specific in cell types
	4	LINE-1 elements prefer low mCW	mainly in brain
	5	Younger LINE-1 element prefer lower mCW	mainly in brain
	6	Loci of Alu and B1 prefers mCW during evolution	Different spots in different cell-types
	7	mCW in enriched in simple repeats	Extremely high in PGC
	8	Intronic region prefer higher mCW on antisense strand	Specific in ES/iPSC
	9	LINE-1 prefer higher mCW on sense strand	Specific in ES
	10	LINE-2 prefer higher mCW on sense strand	Specific in brain and oocyte
mCG	11	Peatks of mCG at promoters of LINE-1	Specific in PGC
